# Rapid Identification of Secondary Structure and Binding Site Residues in an Intrinsically Disordered Protein Segment

**DOI:** 10.3389/fgene.2021.755292

**Published:** 2021-11-02

**Authors:** Soumyanetra Chandra, Gopinath Chattopadhyay, Raghavan Varadarajan

**Affiliations:** Molecular Biophysics Unit, Indian Institute of Science, Bangalore, India

**Keywords:** charged mutational scanning, aspartate, functional residues, interface, protein-protein interactions, protein structure, sequence homology, evolutionary conservation

## Abstract

*Mycobacterium tuberculosis* harbours nine toxin-antitoxin (TA) systems of the MazEF family. MazEF TA modules are of immense importance due to the perceived role of the MazF toxin in *M. tuberculosis* persistence and disease. The MazE antitoxin has a disordered C-terminal domain that binds the toxin, MazF and neutralizes its endoribonuclease activity. However, the structure of most MazEF TA complexes remains unsolved till date, obscuring structural and functional information about the antitoxins. We present a facile method to identify toxin binding residues on the disordered antitoxin. Charged residue scanning mutagenesis was used to screen a yeast surface displayed MazE6 antitoxin library against its purified cognate partner, the MazF6 toxin. Binding residues were deciphered by probing the relative reduction in binding to the ligand by flow cytometry. We have used this to identify putative antitoxin interface residues and local structure attained by the antitoxin upon interaction in the MazEF6 TA system and the same methodology is readily applicable to other intrinsically disordered protein regions.

## Introduction

The biological function of a protein is defined by its structure which in turn is determined by its amino acid sequence (Fischer, 1894). Contrarily, intrinsically disordered proteins involved in protein-protein interaction networks, cellular regulation and signalling execute their function without the need of any defined unique structure (Wright and Dyson, 1999; Dunker et al., 2002), and fluctuate rapidly through a range of conformations. Such conformational states are believed to promote binding to multiple protein partners. Binding to such partners is often accompanied by structuring of the disordered protein or domain through coupled folding mechanisms (Dyson and Wright, 2002). Protein disorder in antitoxin CcdA has been shown to play an important role in binding to cognate toxin, CcdB. Binding occurs at overlapping high and low affinity sites (De Jonge et al., 2009) and this plays a role in rejuvenation of Gyrase bound CcdB by CcdA (Aghera et al., 2020).

Toxin-antitoxin (TA) systems play an important role to combat stress conditions in free living bacteria (Christensen et al., 2003; Pandey and Gerdes, 2005; Hayes and Van Melderen, 2011; Wang and Wood, 2011). Amongst the seven different categories of TA systems, type II is the most abundant and well characterised (Lobato-Márquez et al., 2016). The type II TA systems encode for a labile antitoxin and a stable toxin (Pandey and Gerdes, 2005), which have been shown to play critical roles in plasmid maintenance, adaptive responses to adverse conditions, antibiotic tolerance, persistence and virulence in hosts (Yamaguchi et al., 2011; Maisonneuve and Gerdes, 2014; Wen et al., 2014). Recent studies involving the ten TA modules of *Escherichia coli* have contradicted the correlation between the activation of TA system and persister formation (Harms et al., 2017; Shan et al., 2017; Goormaghtigh et al., 2018; Pontes and Groisman, 2019). However, overexpression of these toxins in *Mycobacterium tuberculosis* have been shown to inhibit growth in a bacteriostatic manner (Gupta, 2009; Ramage et al., 2009; Tiwari et al., 2015; Winther et al., 2016; Agarwal et al., 2018) and contribute to the formation of drug-tolerant persisters (Singh et al., 2010; Sharma et al., 2020). Moreover, significant impairment in survival of *mazF* knockout strains of *M. tuberculosis* has been reported under oxidative stress conditions and in macrophages post infection (Tiwari et al., 2015), suggesting that these TA systems are important for *M. tuberculosis* survival and persistence within the host. There are nine TA systems belonging to the MazEF family in *M. tuberculosis* (Pandey and Gerdes, 2005; Ramage et al., 2009). In existing structures, it is largely the intrinsically disordered C-terminal domain of the MazE antitoxins that binds and neutralises the MazF ribonuclease activity. In addition to the structure of MazEF from *E. coli* (Kamada et al., 2003) and *Bacillus subtilis* (Simanshu et al., 2013), there are only three available crystal structures of full-length MazEF complexes, MazEF4 (Ahn et al., 2017), MazEF7 (Chen et al., 2019), and MazEF9 (Chen et al., 2020) all from *M. tuberculosis*.

In the present study, we have used mutational scanning to rapidly map the binding sites of the intrinsically disordered antitoxin MazE6 and identify residues important for binding its partner MazF6. Based on earlier studies it was observed that it is largely the C-terminal residues and in some cases a few residues of the N-terminus of the antitoxins that are involved in toxin binding (Kamada et al., 2003; Simanshu et al., 2013; Ahn et al., 2017; Chen et al., 2020). There are exceptions to this, such as for HigAB, HicAB, ygiUT systems. However, in all these cases, the toxin gene is before the antitoxin (Budde et al., 2007; Jørgensen et al., 2009; Christensen-Dalsgaars et al., 2010). In such cases, instead of the C-terminus of the antitoxin, the other domains (N-terminal or central domain) are involved in toxin binding (Brown et al., 2009; Yang et al., 2016; Manav et al., 2019). In the case of MazE6, it remains to be formally established that the free antitoxin contains a significant amount of intrinsically disordered regions. However, by analogy with other characterized MazEs (Kamada et al., 2003; Simanshu et al., 2013), it is reasonable to assume that MazE6 also has significant IDP content.

Previous studies in globular proteins have shown that Aspartate scanning mutagenesis is a useful probe of residual burial in proteins (Bajaj et al., 2005). An extended analysis of scanning mutagenesis data from multiple protein systems, revealed the difficulty of distinguishing between buried and active-site residues in globular proteins, purely from phenotypic data on protein function, as both classes of residues are affected by mutation (Bhasin and Varadarajan, 2021). In a recent study, we show that by combining mutational effects on expression and binding/function, it is possible to distinguish between these two classes of residues (Ahmed et al., 2021). The problem, is in principle simpler for IDPs, where most substitutions are not expected to affect expression. Binding is maximally affected at those sites which become buried on partner binding. Prior mutational data with globular proteins showed aspartate followed by arginine to be the best mutagenic probe of residue burial (Bajaj et al., 2005; Tripathi et al., 2016; Ahmed et al., 2021). We therefore used this approach to identify binding interface residues in the case of the MazE6 antitoxin. In the current work, we have used aspartate scanning mutagenesis to mutate the last fifty two residues of the MazE6 antitoxin to probe its interface with MazF6. This method involves screening of purified cognate toxin MazF6 against a panel of single-site aspartate mutants of the interacting partner MazE6 antitoxin, displayed on the yeast cell surface. The binding residues were deciphered by probing the loss of binding of the displayed mutant protein with its cognate partner by flow cytometry. Further, we have mutated selected residues to arginine, to compare the effects of oppositely charged substitutions on toxin binding. We have used the difference in binding signals to further predict the helicity of the antitoxin attained upon binding. We have also compared the effect of substitutions at particular positions with the evolutionary conservation scores at that position. Finally, we have experimentally determined the binding energetics of toxin binding for a small set of MazE6 variants. We have also shown that apparent mutational effects on binding affinities can be easily quantified using our method.

## Materials and Methods

### Plasmids and Host Strains

The *mazE6* antitoxin gene was cloned in the pPNLS yeast shuttle vector, fused to Aga-2p yeast surface protein, under control of the Gal 1-10 promoter to express protein on the yeast cell surface. The *mazF6* toxin gene was cloned in the pET-15b vector for protein expression and purification. EBY100 strain of *Saccharomyces cerevisiae* was used for the yeast cell surface display of the MazE6 protein and *Escherichia coli* host strain BL21 (DE3) pLysE was used for the expression of the MazF6 toxin.

### Cloning and Mutagenesis of the *mazE6* and *mazF6* Genes

Codon optimized genes for the *mazEF6* TA system were PCR amplified from the pET Duet-1 vector, which was synthesized from GenScript (United States). PCR amplified products were gel purified and *in vitro* recombined using Gibson assembly with either pET-15b vector for MazF6 protein purification, or pPNLS vector for MazE6 yeast surface display (Gibson et al., 2009). Recombined products were transformed into *E. coli* and plasmid identities were confirmed by Sanger sequencing.

For generation of mutants of MazE6 protein, the *mazE6* gene was amplified in two fragments with the desired point mutations. The fragments had overlapping regions (introduced during PCR) of 25-30 nucleotides, which were then recombined *in vivo* with pPNLS vector in EBY100 strain of *Saccharomyces cerevisiae*. We have used GAC and CGC codons (most common D and R codons in *M. tuberculosis* H37Rv genome) for all Asp (D) and Arg (R) mutagenesis respectively. We have individually cloned the WT and all the Asp and Arg mutants of MazE6 by setting up individual reactions for each clone. Amplification was done using Phusion Polymerase from NEB as per the manufacturer’s protocol. Plasmid was isolated and PCR amplified, and mutations were confirmed by Sanger sequencing. Upon Sanger sequencing, 43 aspartate and 27 arginine mutants were sequence confirmed, which were then used in this study.

### MazF6 Protein Expression and Purification

The MazF6 toxin was purified as described previously (Sharma et al., 2020). Briefly, cultures were grown in TB media, induced with 1.0 mM IPTG at an OD of 0.6 for 7 h at 20°C for toxin (His)_6_MazF6(FLAG)_3_ expression. The pellet was re-suspended in re-suspension buffer pH 8.0 (10 mM HEPES, 100 mM NaCl, 100 mM Arginine containing Protease inhibitor Tablet, Roche) and sonicated. The supernatant following centrifugation was incubated with 2 ml Ni Sepharose 6 Fast Flow (GE Healthcare) for 4 h at 4°C. Following two column volumes wash with wash buffer (10 mM HEPES, 100 mM NaCl, 100 mM Arginine and 50 mM imidazole, pH 8.0), the MazF6 was eluted with 1 ml of elution buffer (10 mM HEPES, 100 mM NaCl, 100 mM Arginine and a gradient of imidazole (100–900 mM), pH 8.0). The protein was stored in storage buffer (10 mM HEPES, 100 mM NaCl, 100 mM Arginine, and 500 mM imidazole, pH 8.0) at -80°C after concentration.

### Binding Studies of MazE6 Antitoxin With MazF6 Toxin Using Yeast Surface Display Coupled to Flow Cytometry

The binding of intrinsically disordered MazE6 antitoxin with MazF6 toxin was measured using YSD. The full-length MazE6 antitoxin was expressed on the yeast cell surface and the level of expression was measured using anti-HA antibody and goat anti-chicken conjugated to Alexa fluor 488 incubated with the yeast cell surface displayed protein as explained earlier (Najar et al., 2017; Ahmed et al., 2021). Binding was measured by incubating the yeast cell expressing full-length MazE6 with 1 nM of its cognate partner MazF6 toxin, having 3X FLAG tag. The bound protein amount was detected by the anti-FLAG antibody (1:300 dilution) and rabbit anti mouse conjugated to Alexa fluor 633 secondary antibody (1:1600 dilution). In all binding studies, the dimeric concentration of the MazF6 protein is used. The voltage settings used for Forward Scatter (FSC), Side Scatter (SSC), Alexa 488, Alexa 633 are 143, 247, 467, 687 respectively. An FSC threshold of 5000 is used for event collection. First a FSCA vs SSCA plot is used for selecting the desired yeast populations. This is followed by doublet elimination using FSC area vs FSC width and SSC area vs SSC width plots. Finally, the minimum and maximum autofluorescence values used for uninduced cells for Alexa 488 and Alexa 633 are −99, 610 and −276, 1315 respectively. Mean fluorescence value was calculated by analysing 10,000 cells for WT and each mutant. The binding studies were carried out on a BD Aria III FACS machine, using BD FACSDiva Software Version 6.13. All the mean fluorescence intensities used in the study are directly provided by the BD FACSDiva software at the time of analysis.

### Calculation of Expression and Binding Activity Scores for MazE6 Single Mutants

The Alexa fluor-488 fluorescence intensity was used to compare surface expression of wildtype and variant MazE6 proteins. Mean Fluorescence Intensity (MFI) of expression (Mean Alexa fluor-488 fluorescence intensity for all cells analysed for each sample) for mutants were normalised with respect to that of the WT, to obtain MFI^exp^, using formula:

MFI^exp^ = Expression MFI of mutant/Expression MFI of WT Equation 1, which enables to estimate the relative extent of surface expression of the MazE6 variants on yeast cells.

To accurately estimate and compare toxin binding activities of MazE6 variants, it is important to account not only the binding signal (Alexa 633), but also the differences in surface expression upon substitution. Therefore, we used Alexa 633: Alexa 488 ratio fluorescence intensity ratio recorded during FACS analysis of surface displayed MazE6 proteins. Mean Fluorescence Intensity of binding: expression was used to score mutants based on their toxin binding affinity. For comparisons across experiments we have normalised the ratio of fluorescence intensities for mutants with respect to that of WT as given below:

MFI^ratio^ = Mean Alexa 633: Alexa 488 ratio Fluorescence Intensity of mutant / Mean Alexa 633: Alexa 488 ratio Fluorescence Intensity of WT Equation 2.

The normalized MFI^ratio^ scores (with respect to WT) were calculated in two biological replicates and the mean was used in our work. The MFI^ratio^ values in replicates, mean and standard deviation are provided in Supplementary Table S1.

### Prediction of Helical Structural Features From Mutational Data

The MFI^ratio^ values for aspartate mutants were averaged over a window of five residues to obtain MFI^avg^ which was then subtracted from the MFI^ratio^ values to obtain the corrected MFI^ratio^ values. These values were fitted to a simple sinusoidal curve,

y=a sin(2πx/b+c)

where *π* = 3.14, a = amplitude, b = periodicity and c = phase. For the four residues (L38, I57, A65, and N70) for which we have no Asp mutational data, we have used WT values.

### Conservation Score Calculations

The evolutionary conservation score was calculated from the online server ConSurf which used multiple alignment of homologous sequences to build a phylogenetic tree. It also provided the frequencies of all amino acid residues at each position by comparing all homologs. The minimum and maximum percentage identity cut-off used was 20 and 95% respectively for the multiple sequence alignment of the antitoxin MazE6.

### Quantitative Estimation of K_d_ for WT MazE6 and Single Mutants

WT and eight single mutants of MazE6 cloned in the pPNLS yeast surface display (YSD) vector were used to quantify the binding energetics for interaction with cognate toxin, MazF6. We investigated toxin binding of surface displayed MazE6 at varying concentrations of purified MazF6 protein ranging from 35 fM to 500 nM. The mean MFI of binding as a function of ln [MazE6] was fit to the one site ligand binding model using SigmaPlot 14, based on previously described binding affinity calculation using YSD based titration (Rathore et al., 2017). The equation used for fitting to one site ligand binding is:

$$\mathrm{y=}\left(B_{\max}+\left[L\right]\right)/\left(K_{d}+\left[L\right]\right)$$

Where *y* is the binding fluorescence intensity, B_max_ = the maximum value of *y*, [L] = concentration of ligand, MazF6 protein, and K_d_ = dissociation constant.

In case of the K_d_ estimations, we have carried out titrations of the WT MazE6 construct at 16 different concentrations of MazF6 protein, in two technical replicates. The standard deviation of WT K_d_ of the two independent experiments is 0.19 nM (∼20% Standard Deviation). Previous work in our lab suggests K_d_ determined by the yeast surface based titration method used here to be quite robust, showing low variation (<10% Standard Deviation) among replicates (Rathore et al., 2017). Therefore, we did not carry out the titrations for mutant MazE6 constructs in replicates.

## Results

### Investigation of Mutational Effects on Partner Binding in the C-Terminal Region of MazE Antitoxin

**FIGURE 1:**
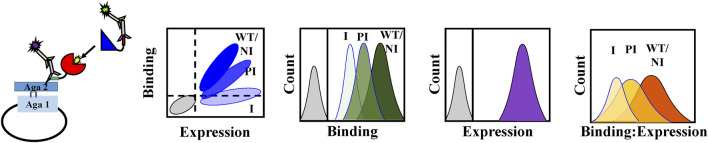
Schematic representation of the rapid mapping of the binding site of a natively unfolded antitoxin through mutational scanning. Aspartate and arginine mutants of MazE6 are introduced at the predicted ligand binding interface (31-82 residue stretch). The mutant is displayed as a fusion protein on the yeast cell surface and its binding to the cognate partner is observed by flow cytometry. Introduction of a mutation in the ligand binding site in the MazE6 protein may generate different populations, depending on the location of mutation. Non-interacting residues marked as NI will not change binding upon mutation, similar to WT. However, both partially interacting residues marked as PI and interacting residues marked as I will result in a reduced binding upon mutations, with the hot spot interacting residues showing the maximum effect on binding. To estimate the relative toxin binding affinity for MazE6 variants we used mutational scores based on binding: expression fluorescence intensities, namely MFI^ratio^.

MazE6 (Uniprot ID: P9WJ87; Locus name: Rv 1991A; Synonym: mazE-mt3) is an 82 residue protein antitoxin encoded by the *M. tuberculosis* TA operon *mazEF6* (mt3), that is not well characterized. MazF6 (Uniprot ID: P9WII3; Locus name: Rv 1991c; Synonym: mazF-mt3) toxin has been shown to have distinct sequence specific endoribonuclease activity that prefers 5’ UU↓CCU 3’ and 5’ CU↓CCU 3’ for mRNA cleavage (Zhu et al., 2008), which can only be prevented by direct interaction with its cognate antitoxin MazE6 and not cross-reaction with non-cognate MazE antitoxin molecules (Ramirez et al., 2013). The MazF6 mRNA degrading activity has been shown to have bacteriostatic effects and is associated with drug tolerance (Tiwari et al., 2015). Structural information regarding the MazE6 antitoxin in its free and MazF6 toxin bound state is currently unavailable. Although MazE6 shows low sequence similarity to its homologs, we have attempted to model the MazE6 and MazEF6 complex structures using the SWISS-MODEL server (Waterhouse et al., 2018), based on the available homologous MazEF complex structures. Modelling using the *Bacillus subtilis* MazEF structure (PDB ID: 4ME7, (Simanshu et al., 2013)) suggests that only the residues 58 to 78 of MazE6 associate with the toxin MazF6 (Supplementary Figure S1A). Comparative modelling using the Robetta server also yields an unreliable structural model of the MazEF6 complex comprising of single monomers of the toxin and antitoxin chains (Supplementary Figure S1B). The predictions from a homology based model for MazEF6 (Tandon et al., 2020) also correlated only moderately with the present experimental data for the interface residues involved in toxin-antitoxin interaction (Supplementary Figure S1C). Comparing functional annotations and available structures of antitoxins of type II CcdAB, VapBC, and MazEF TA complexes reveals a characteristic well-structured N-terminal dimerization domain and a C-terminal intrinsically disordered domain that interacts with the cognate toxin and forms an extended bent helical structure upon binding. The C-terminal toxin binding domain typically encompasses the last thirty-five to fifty residues in type II antitoxins. To map the unknown MazF6 toxin binding sites of MazE6 antitoxin, we carried out single site mutagenesis at C-terminal residues 31-82 in the MazE6 protein and experimentally probed the mutant binding activities. Site directed mutagenesis has been previously attempted to identify functional residues in TA pairs (Tandon et al., 2019). However, such studies are restricted by limitations associated with rational designing of mutants at putative interface residues for experimental characterization. We have previously shown that Asp scanning mutagenesis can be used to predict residue burial in the context of globular proteins (Bajaj et al., 2005). In case of IDPs, we do not expect introduction of a charged residue to grossly perturb the structure or stability of the unbound IDP. However, if a binding site residue is replaced by a charged residue (or oppositely charged if the WT residue is charged), we predict a major perturbation in the free energy of binding. We have therefore employed yeast surface display to investigate functional consequences of systematic aspartate (Asp, D) and a few arginine (Arg, R) substitutions at the 31-82 residue stretch of MazE6 antitoxin (Figure 1).

**FIGURE 2:**
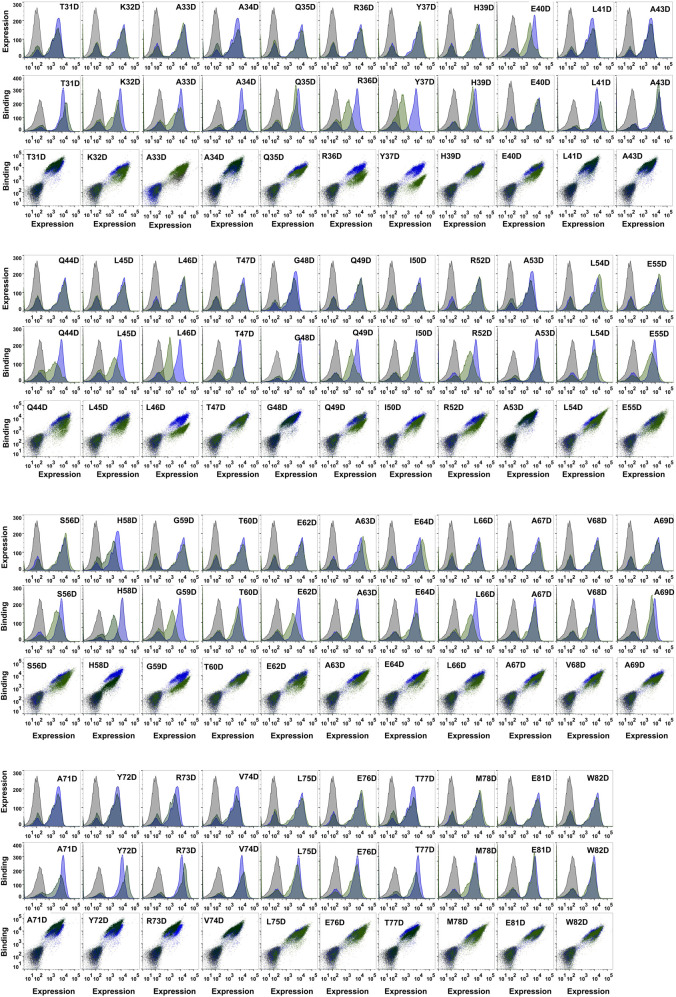
Analysis of expression and MazF6 binding of different MazE6 aspartate mutants. Comparison of the surface expression and MazF6 binding of MazE6 aspartate mutants with that of the WT MazE6 on the yeast cell surface are shown. WT MazE6 histogram (blue) is overlaid with the histograms obtained for the mutants (green) in each of the plots. The uninduced cells are shown in grey.

**FIGURE 3:**
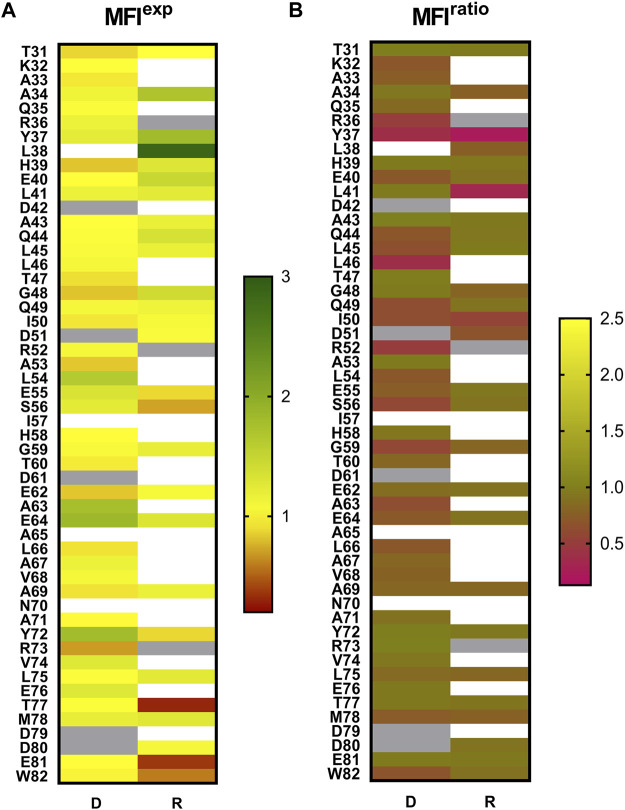
Mutational effects in MazE6 depicted as heatmaps. **(A)** The normalised expression scores of mutants, MFI^exp^ = expression MFI(mutant)/ expression MFI(WT). The MFI^exp^ scores describe the expression levels of MazE6 proteins on yeast cells with respect to WT. **(B)** Normalised MFI^ratio^ = binding: expression ratio MFI (mutant) / binding: expression ratio MFI (WT), where binding: expression ratio MFI is the mean Alexa 633:Alexa 488 fluorescence intensity ratio, illustrated in heatmap for the investigated MazE6 substitutions to aspartate and arginine. The MFI^ratio^ scores help distinguish the interaction of surface displayed MazE6 mutants to purified WT MazF6, with respect to the WT construct of MazE6. The mean of two replicates has been plotted here in the heatmap. The blank cells indicate mutants where the mutant was not made. Grey cells indicate WT residues. In all cases, normalization is with respect to WT values.

The wild-type and mutant MazE6 molecules with N-terminal HA tags were displayed on the yeast cell surface as a C-terminal fusion to the yeast surface protein Aga-2p. The surface expression and binding to purified and 3X-FLAG tagged cognate toxin partner, MazF6 protein, was monitored for each variant individually by fluorescence activated cell sorting (FACS) using anti-HA antibody and anti-FLAG antibody to detect surface expression and binding respectively, along with suitable fluorophore tagged secondary antibodies. We observed that the majority of single site charged substitutions had limited effects on the surface expression of the variant protein molecules in MazE6 (Figures 2, 3A). To quantify MazF6 toxin binding activity of single mutants with respect to wildtype MazE6, we used, the mean binding: expression fluorescence intensity ratio (bind:exp MFI ratio, denoted as MFI^ratio^) (Figure 1). Lower binding activity of the mutant denoted by lower mean fluorescence intensity with respect to WT values, specifies higher sensitivity to mutation (Figure 3B). The sensitivity to charged substitutions in MazE6 helps identify the residue specific contributions in MazE6 to binding of its cognate toxin MazF6.

### Inferences About Toxin Interaction Sites From MazE6 Mutagenesis Data

We systematically mutated each residue in the MazE6 31-82 stretch (except the WT Asp residues) to aspartate and probed the corresponding toxin binding activity (Figure 2). We also investigated twenty-seven randomly chosen arginine single variants of MazE6 (Supplementary Figure S2). Our study suggests that while single substitutions reduce the antitoxin affinity to the cognate toxin, they fail to completely abolish binding (Figure 3B). This is owing to the significantly large interface involved in typical TA interactions. However, small binding affinity changes are efficiently detected by binding: expression MFI^ratio^. For ease of comparison across experiments, we have used normalized mutant MFI^ratio^ values with respect to that of WT. Wildtype MazE6 therefore has an MFI^ratio^ value of 1. Mutants displaying lower MFI^ratio^ values can be inferred to be binding defective and thus functionally important for interaction with the toxin. Analysis of statistical k-means clustering of aspartate mutational effects in MazE6 into two classes, yields class-1 (similar to WT) and class-2 (lower binding affinity) having mean MFI^ratio^ scores of 0.89 (standard deviation, *σ* = 0.07) and 0.58 (standard deviation, *σ* = 0.11) respectively. Based on these identified classes, we categorize mutants having MFI^ratio^ lower than 0.82 (= mean^class1^—σ^class−1^) as binding defective (for either Asp or Arg substitutions). Using the criteria stated above, we identified twenty-three residues in the fifty-two residue long stretch (residues 31-82) examined, to contribute to toxin binding activity. These are K32, A33, R36, Y37, L38, E40, Q44, L45, L46, Q49, I50, D51, R52, L54, S56, G59, A63, E64, L66, A67, V68, A69, and M78. While most of these residues can be confidently referred to as interface residues, there may be a few non-interacting residues that show low mutational tolerance upon substitutions by virtue of impeding vicinal interactions and can thus be erroneously predicted as interacting. For example, Glycine residues that adopt positive Φ values inaccessible to other residues, might be expected to show high sensitivity to mutations. Of the two glycine residues found in the MazE6 C-terminal domain, one (G59) shows low tolerance to charged substitutions (Figure 3B). This glycine could be an interacting residue or an interface shaping residue with a helix-breaking positive Φ backbone torsional value, as observed in case of glycine residues residing at helix terminating positions in several type II antitoxins. However, when substituted to a non-charged cysteine residue, the observed MFI^ratio^ was 0.8, indicating no significant difference in binding. Therefore, we conclude that MazE6- G59 does not seem to adopt a positive Φ value, unlike the situation for several Gly residues in other antitoxins.

**FIGURE 4:**
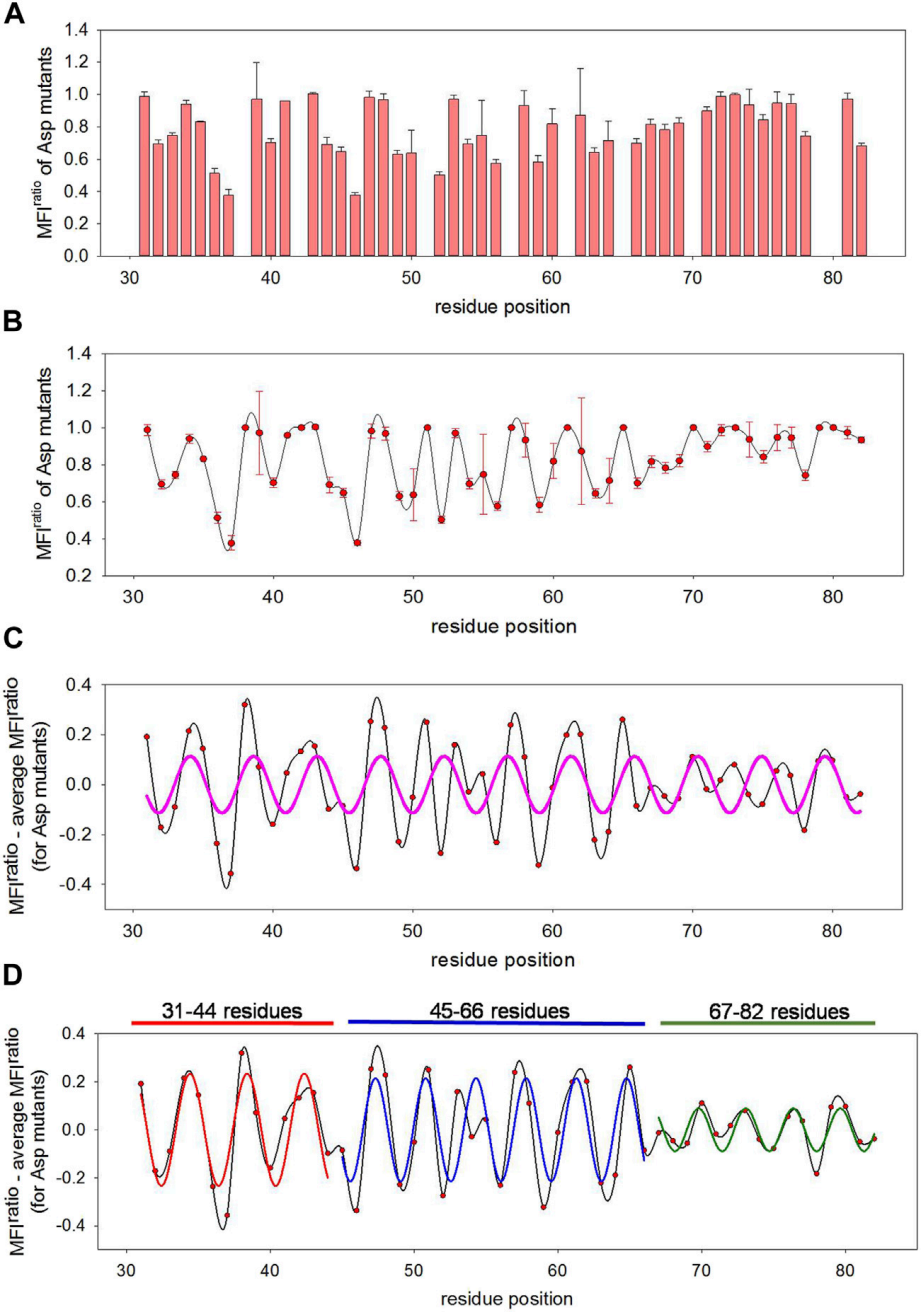
Predicting local structural features attained by disordered MazE6 upon toxin binding. **(A)** MFI^ratio^ of aspartate mutants show non-uniform distribution of mutational effects across the protein length. The mean values of two biological replicates are plotted here. The error bars describe the standard deviations between two replicates. **(B)** MFI^ratio^ of aspartate mutants (mean values in red circle, with standard deviations depicted as error bars) as a function of residue positions plotted as a spline curve (shown in black), that reveals an oscillating pattern in MazE6 mutational effects. **(C)** MFI^ratio^-MFI^avg^ fit to a sinusoidal curve. The MFI^avg^ is the MFI^ratio^ of aspartate mutants averaged over five residue windows. When a single sinusoidal curve is fit to the corrected mutational scores of residues 31-82, the fit (shown in pink) is poor. **(D)** MFI^ratio^-MFI^avg^ for residue stretches 31-44, 45-66 and 67-82 fit to three separate sinusoidal waves. The fits for 31-44, 45-66 and 67-82 residue stretches are shown in red, blue and green lines respectively.

Surprisingly, we found the overall mutational sensitivity in the 31-66 residue stretch to be significantly higher than that at the C-terminus (Figures 3B, 4A). The 31-45 residues were not expected to be specifically involved in the toxin interaction, based on the general structural and functional features of type II antitoxins, where the first forty residues of antitoxin generally form the N-terminal DNA interacting domain and the C-terminal half is primarily involved in toxin binding. Interestingly, a recent crystal structure of the homologous *M. tuberculosis* MazEF9 (mt 1) complex structure (PDB ID: 6KYT) reveals that while residues 1-40 of the 82 residue MazE9 antitoxin form the structured N-terminal dimerization domain, certain residues in this domain also interact with toxin molecules (Supplementary Figure S3). The present mutational data for MazE6 also suggest the involvement of the N-terminal domain of the MazE6 antitoxin in interaction with its cognate toxin.

### Salient Features of Interface Residues in Type II Antitoxins

Low hydrophobicity and presence of charged residues has been implicated in promoting disorder in proteins (Uversky et al., 2000). We however find both hydrophobic as well as charged residues to be prevalent in MazE6, most of which are found to be functionally important (Figure 3B). Aliphatic residues are highly prevalent in MazE6 and are inferred to contribute substantially to MazF6 binding in the MazE6 antitoxin. Experimentally determined, functionally important residues in the C-terminal stretch of 60-82 are largely aliphatic in nature (Figure 3B).

Since the toxin is a ribonuclease, it is predominantly expected to have basic residues in its active-site and is expected to be neutralized by an antitoxin with compensatory acidic residues. However, this is always not the case. In the case of solved crystal structures of MazEF from *Bacillus subtilis* and MazEF4 and VapBC30 from *M. tuberculosis*, the antitoxins have large numbers of basic residues which are also involved in the toxin binding. Further, for other antitoxins such as the MazE1, MazE2, MazE3, MazE8 of the MazEF TA systems from *M. tuberculosis*, for which the crystal structure of the complex is not available, we observe from the sequence information, that the C-terminal regions of the antitoxins have large numbers of basic residues. In the present study, MazE6 shows a high frequency of the acidic residues Aspartate and Glutamate, which upon mutation to oppositely charged, basic arginine residue generally do not show drastic effects on toxin binding (Supplementary Figure S4). In order to probe effects of opposite charged substitutions in MazE6, we examined Arginine substitutions at WT residues that were E or D. Unfortunately, for 3 of the 5 WT Asp positions (D42, D61, and D79) and 1 of the 6 WT Glu positions (E76), we could not successfully clone Arginine mutants despite multiple attempts. For the two Asp to Arg mutants (D51R and D80R) that we could probe, we observed D51R binding to be slightly affected while D80R binding was unaffected. For Glu to Arg mutants, (E40R, E55R, E62R, E64R, and E81R) none of the mutants were observed to show binding defects (Supplementary Figure S4). It appears that unlike the case of positively charged WT positions where mutations to D largely hinder binding, the substitutions of negatively charged WT positions to R show position specific and typically smaller effects on binding in MazE6. This demonstrates that while an overall negative charge is preferred by the antitoxin molecule, loss of a single negative charge bearing side group fails to impact overall toxin interactions. In contrast, the rarer positively charged arginine and lysine residues appear to be important for binding, as Asp substitutions at these positions diminish toxin binding (Supplementary Figure S4A). At positions bearing uncharged WT residues, substitutions to arginine hampered toxin binding more severely compared to the substitutions to aspartate (Figure 3B). This suggests that introduction of positive charge bearing side groups destabilise the toxin binding event by altering the overall charge distribution of the MazE6 antitoxin molecule. Consistent with these observations, the MazEF6 complex modeled by SWISS-MODEL shows an overall negatively charged surface of MazE6 antitoxin interacting with a positively charged binding cleft on the MazF6 toxin dimer (Supplementary Figure S4B). Charge distribution of the antitoxins and the complementary binding cleft of cognate toxins in the homologous MazEF systems are identified to be oppositely charged, with the antitoxin molecule typically displaying an acidic surface in most cases (Supplementary Figure S3).

### Prediction of Local Secondary Structural Features From Mutational Effects in MazE6 Aspartate Variants

Mutational tolerance upon aspartate substitutions in MazE6 across the length of the C-terminal fifty-two residues under study display an oscillating pattern (Figures 4A,B). However, the severity of mutational effects is irregular across the length indicating the presence of unequal contributions of different regions to toxin binding (Figures 3B, 4A). To remove the non-uniform region specific contribution to binding, we subtracted the MFI^ratio^ values by the values averaged over a five residue window (Figure 4) as described earlier (Newberry et al., 2020). We have previously tested this correction, with 5, 7, 9, and 11 residue window averages. Upon fitting these corrected data to sinusoids, we found identical local periodicity for the all the 5, 7, 9, and 11 residue window calculations. We therefore chose to use 5 residue window average, since it allows for an easier identification of possible phase changes in the waveform. Identification of such discontinuity is essential in correctly predicting structures of IDPs in their partner bound form, where in many cases the IDPs wrap around the globular partner protein upon binding and there are drastic angular changes in the protein backbone. When fitted to a sinusoidal curve, the corrected aspartate mutational MFI^ratio^ for residues 31-82 shows a poor fit (Figure 4C). The pattern suggested a possible phase change in the wave-like pattern in the mutational effects at residue positions 44-45 and overall low amplitude in the 66-82 residue stretch. We therefore fitted residue stretches 31-44, 45-66, and 67-82 to separate sinusoidal waveforms. The three stretches fitted to individual sinusoidal waves with periodicities of 4 ± 0.9, 3.49 ± 0.6, and 3.28 ± 0.6 respectively and with R values of 0.88, 0.73, and 0.81 respectively (Figure 4D). We can therefore infer with high confidence that the MazE6 residues 45-66 form a canonical *α*-helical structure, as *α*-helices typically have 3.6 amino acid residues per turn. Residues 67-82 are also likely to form an *α*-helix as the periodicity is close to 3.3 residues. It is likely that the residues 45-82 form a continuous helix and the change in the oscillating pattern observed at the residue 66 is owing to the observed low contribution of the MazE6 C-terminus residues 67-82 to toxin binding (Figures 4D, 3B). The residues 31-44 likely either have an irregular structure or presence of helical features that may be masked by an uneven binding interface, where the toxin partner wraps around the helical antitoxin thus distorting the observed periodicity. The IDP segments are commonly found to form extended helical structures with one face of the helix interacting with the protein partner. Such structural organization in extended helices in IDPs allows facile elucidation of structural features from mutational scanning, using the approach outlined here.

### Quantification of Binding Energetics of MazF6 Binding in MazE6 Variants

**FIGURE 5:**
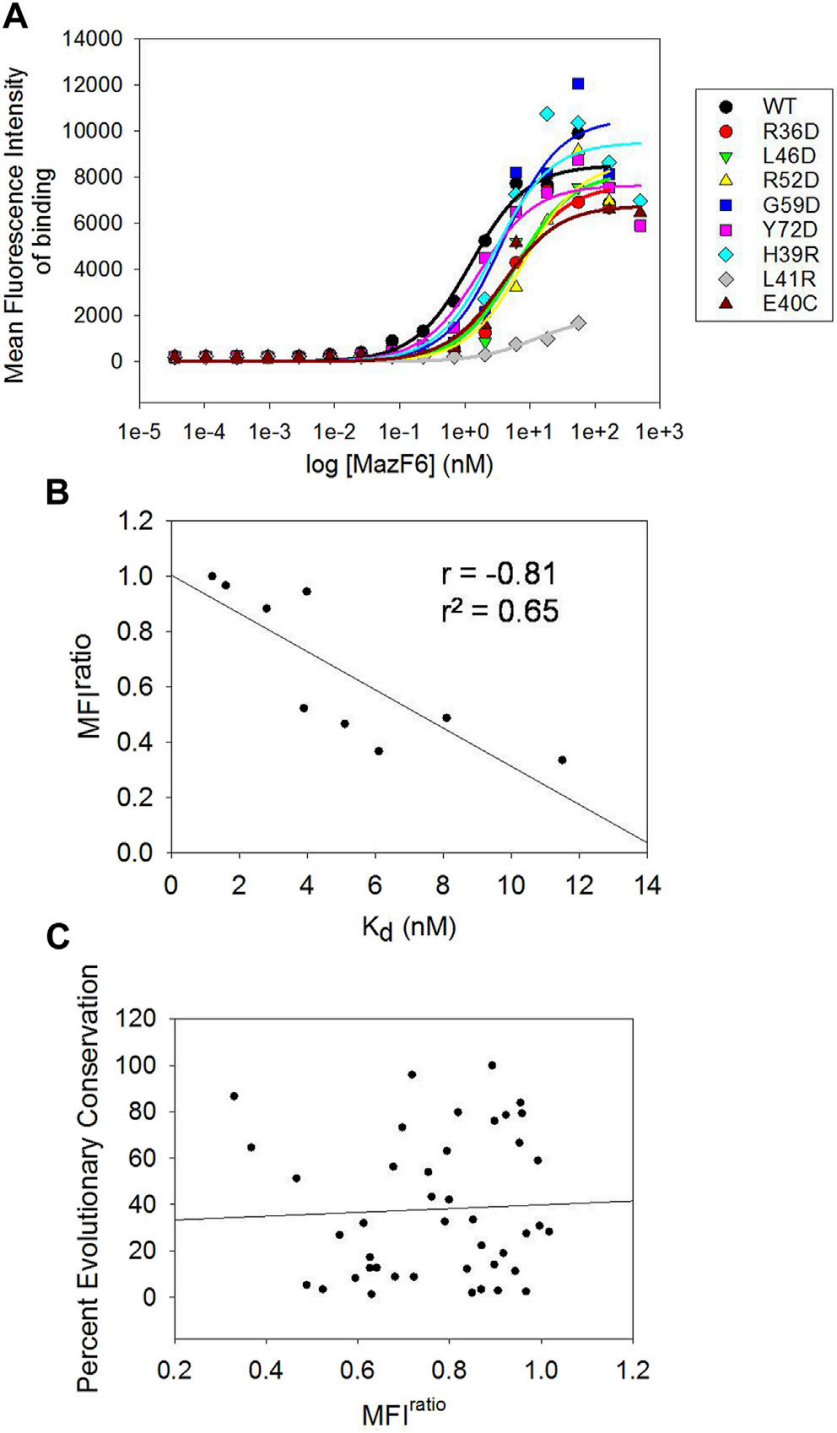
Experimental quantification of K_d_ of binding, and correlation of evolutionary conservation with mutational effects **(A)** Mean Fluorescence Intensity (MFI) of toxin binding as a function of MazF6 concentration (nM) experimentally recorded for MazE6 WT and eight single mutants. The traces were used to obtain K_d_ values (see Methods). **(B)** Correlation between the MFI^ratio^ scores from individual FACS analyses of single mutants with the experimentally determined K_d_ values from titration studies. **(C)** Correlation of MFI^ratio^ scores of aspartate mutants at each position in the 31-82 stretch of MazE6 with the respective percent evolutionary conservation score.

The MFI^ratio^ scores derived from the individual FACS analysis of MazE6 single site mutants are useful as arbitrary scores that help to distinguish differences in MazF6 binding affinities amongst the variants. To validate the MFI scores, we have also experimentally determined the dissociation constants (K_d_) of MazE6-MazF6 interaction for WT and a small set of single site mutants of MazE6. Yeast surface displayed MazE6 was titrated against a range of different dimeric concentrations (35 fM—500 nM) of purified C-terminal 3X-FLAG tag bearing MazF6 protein. The mean fluorescence intensities of binding (Alexa fluor-633 intensity) were then fit to a one-site binding model to obtain the dissociation constants for each variant MazE6 molecule (Figure 5A), in order only to examine the correlation with MFI^ratio^ values. From the available structures of the MazEF complexes, it is known that each monomer of the antitoxin dimer binds to a different toxin dimer, and the complexes form C2 symmetric structures. Thus, it is expected that the binding of the antitoxin at each toxin dimer is independent. In case of two or more distinct binding events with different affinities, two (or more) steps of binding is expected to be observed in titration studies. Despite using a substantial range of 16 MazF6 concentrations (ranging from 35 fM to 500 nM) in each experiment, all our data is consistent with identical and independent sites.

When correlated with the MFI^ratio^ scores, the experimental K_d_ values show consistently good correlations (Figure 5B). This indicates that the Binding: Expression MFI values obtained from the FACS analysis provide a good qualitative estimate of the relative dissociation constants of protein-protein interactions under study.

### Poor Correlation of Mutational Tolerance With Evolutionary Conservation

Prediction of functional consequences upon mutations often relies on evolutionary conservation information derived from comparing homologous protein sequences. Positions showing highly conserved residues generally indicate a functional role of the residue. Surprisingly however, interfacial residues inferred from the current scanning mutagenesis experiments show poor correlation with evolutionary conservation (Figure 5C), revealing that residues conserved across homologous MazE antitoxins do not necessarily contribute to binding and vice versa. The percent occurrence of aspartate and arginine residue at each residue position is also uncorrelated with our scanning mutagenesis results. Multiple sequence alignments among MazE6 homologous genes obtained using the ConSurf server (Ashkenazy et al., 2016) show low sequence identity and poor conservation in the C-terminal 40-82 stretch (Supplementary Figure S5). Multiple sequence alignment of the MazE6 antitoxin sequence with homologous *E. coli* and *B. subtilis* MazE as well as the *M. tuberculosis* MazE 4, 7, and 9 sequences shows poor residue conservation at inferred functional sites (Supplementary Figure S6A). Moreover, comparing available structures of the antitoxins and the cognate toxins reveal low RMSD values, indicating low structural similarity among homologs in case of the MazEF TA modules (Supplementary Figures S6B–D).

## Discussion

Despite the striking abundance of intrinsic disorder coded by genomes and emerging evidence of association of a plethora of human diseases with protein disorder, mapping of functional residues in disordered proteins remains relatively unexplored. While deep mutational scanning approaches produce a comprehensive mutational landscape, parallel low-throughput yet efficient methods to screen a small number of variants yielding useful structural and functional information are often more practical. We present here a very fast and inexpensive interface mapping approach using systematic aspartate scanning mutagenesis that can be readily applied to investigate partner binding of disordered proteins. We also investigated mutational effects of substitutions to arginine residues at certain positions. Using yeast surface display combined with fluorescence activated cell sorting techniques we characterized the cognate toxin binding activity of single substitutions in the apparently disordered C-terminal 31-82 residue region of *M. tuberculosis* antitoxin MazE6. Examination of mutational tolerance in MazE6 reveals the residue specific contributions to MazF6 binding energetics. Residue positions with low mutational tolerance were inferred to be involved in the toxin-antitoxin binding interface. However, since there is no experimental structure of the complex or reliable models, we could not validate our experimentally inferred interface residues. Surprisingly, mutations at positions 31-45 of MazE6 led to a drastic reduction in toxin binding affinity, when compared to mutations in the C-terminal 67-82 stretch. This is contrary to the structural data in most existing TA complexes where the C-terminal region makes the largest contribution to toxin binding. Based on the repetitive pattern observed in the mutational tolerance in MazE6, we show that periodicity in mutational effects can be used to predict local structures attained by disordered proteins upon partner binding. Based on the fitted periodicity observed in toxin binding activity of mutants across the length of MazE6, we predict a distorted but helical, toxin-interacting 31-45 residue stretch, a canonical *α*-helical and toxin-interacting 45-66 residue stretch and a slightly distorted but near *α*-helical 67-82 residue stretch of MazE6 that contribute moderately to toxin binding. Comparison with available complex structures of MazEF homologs indicate that all the antitoxin structures are unique and also differ from the predicted structural and interfacial features in MazE6 in terms of helical content of C-terminal domain as well as region specific contribution of N- and C-terminus to cognate toxin binding (Supplementary Figure S3). Our Asp scanning mutagenesis therefore reveals a unique toxin-antitoxin interaction module in case of MazEF6 system, where the MazE6 antitoxin is revealed to form a largely helical structure upon binding to the cognate toxin, with the last sixteen residues in the MazE6 C-terminus appearing to be largely redundant for the interaction. Using this methodology, periodicity of stretches involved in interaction with a known partner can be easily determined. We expect that in cases where a *β*-strand forms an extended interactive interface with a partner, these will exhibit an ∼2 residue periodicity in their mutational effects. This remains to be tested.

We have also experimentally determined dissociation constants (K_d_) for MazF6 binding to the WT and a small subset of mutants of MazE6. Our MFI^ratio^ scores show good correlation with the experimental K_d_ values, validating our yeast surface display coupled FACS approach of estimating binding affinities in terms of fluorescence based arbitrary scores, through investigation of individual single mutants. The linear fit of experimental K_d_ values to the MFI^ratio^ scores, also serves as an internal standard allowing easy quantification of apparent K_d_ values for mutants scored based on MFI^ratio^ values.

The MazEF6 models generated by several computational approaches used in this study failed to accurately predict the MazF6 binding interface residues of MazE6 antitoxin. Several interface residues identified experimentally were either not modeled, or did not appear to be interacting with toxin in the model of the complex (Supplementary Figure S1). The availability of more TA complex structures and corresponding mutational data will help refine local structure predictions from mutational effects. In the present study we examined the 31-82 stretch of MazE6 because for the majority of antitoxins, the N-terminal region is involved in DNA rather than toxin binding. However, the mutational data obtained in this study, suggest that the N-terminal stretch of residues 31-82 is important for toxin binding. Hence in future studies, we will also probe the role of the remaining N-terminal residues from 1-30 in MazE6. There is a poor correlation of mutational effects on toxin binding with evolutionary conservation. This further suggests that the toxin binding activity is not the sole contributor to the selection pressure on MazE primary sequence during the process of evolution. Conservation of disorder in the polypeptide chain and promiscuous binding to several non-cognate partners could also be possible forces driving evolutionary selection of residues (Zhu et al., 2010). MazE6 antitoxin shows low overall sequence and structural similarity with its homologs as is also observed in case of the cognate MazF6 toxin, therefore, making it difficult to predict reliable structures through homology modelling, hence requiring alternate approaches. Low sequence similarity in both the binding partners could hint at the possibility of toxin-antitoxin interactions unique to each homologue and possibility of coevolution of TA pairs, causing divergence of sequences in both toxin and the antitoxin sequences (Aakre et al., 2015). However, because of the limited sequence data, and large sequence diversity of MazEF orthologs and paralogs, it is challenging to identify signatures of co-evolution in the MSAs and to use such information to identify inter-residue contacts. Moreover, it is interesting that residues found in the wildtype sequence of MazE6 are often not the most common residue to be found at that position as inferred by multiple sequence alignment analysis of MazE homologs. These studies demonstrate the shortcomings of using evolutionary conservation to infer functionally important residues, or in prediction of mutational effects on protein activity and fitness in the specific case of TA systems.

## Data Availability

The original contributions presented in the study are included in the article/Supplementary Material, further inquiries can be directed to the corresponding author.
