# Supplementary material for: Rapid Identification of Secondary Structure and Binding Site Residues in an Intrinsically Disordered Protein Segment
Source: Front Genet. 2021 Nov 2;12:755292. doi: 10.3389/fgene.2021.755292 (PMC8593223; doi:10.3389/fgene.2021.755292)
Supplement: Supplementary file 1 [file DataSheet1.pdf]

Supplementary Figures

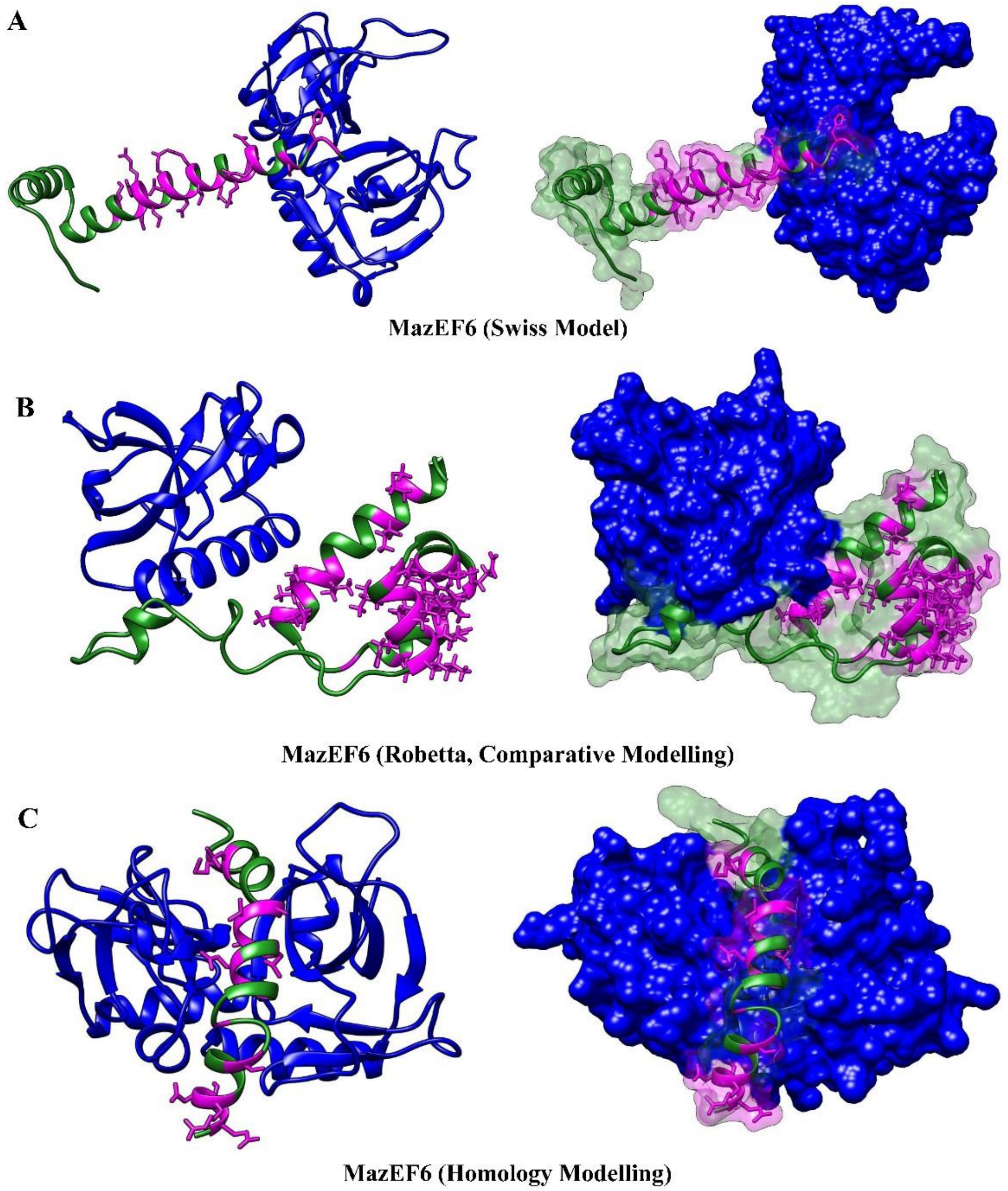

Figure S1. Comparison of MazEF6 complex structure modelled by several computational predictors. Comparison of model structure of MazEF6 (A) as predicted by SWISS-MODEL (Waterhouse et al., 2018), (B) Robetta (Kim et al., 2004) and (C) Homology modelling (Tandon et al. 2020). The right panel shows the surface representation. The experimentally obtained interacting residues are mapped on the complex structure in magenta. In all cases we observe, the inability of the current available predictors to accurately determine structures of either the antitoxin alone or the hetero-oligomeric complexes.

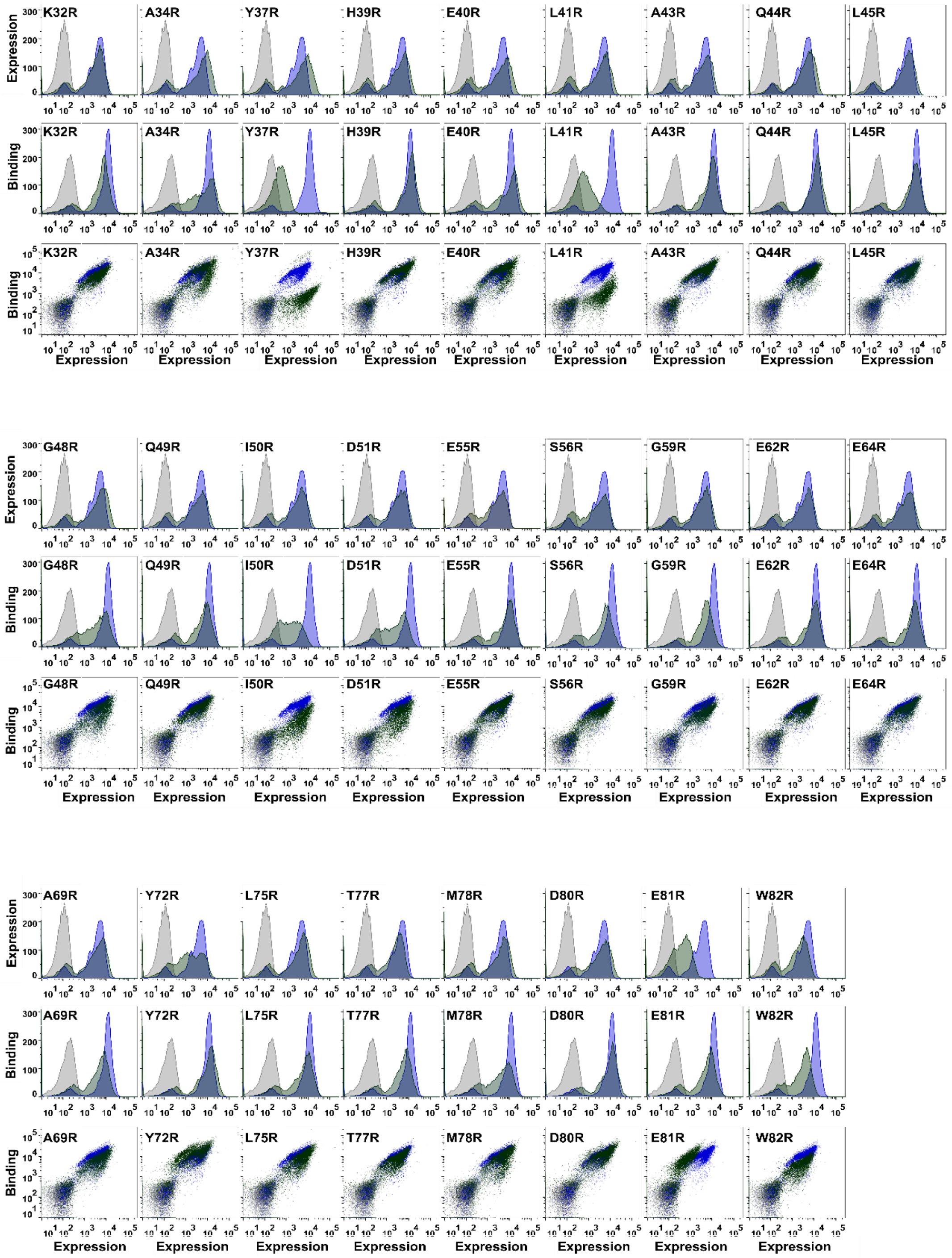

Figure S2. Analysis of expression and MazF6 binding of different MazE6 arginine mutants. Comparison of the expression and MazF6 binding of the MazE6 arginine mutants with that of the WT MazE6 on the yeast cell surface are shown. WT MazE6 histogram (blue) is overlaid with the histograms obtained for the mutants (green) in each of the plots. The uninduced cells are shown in grey.

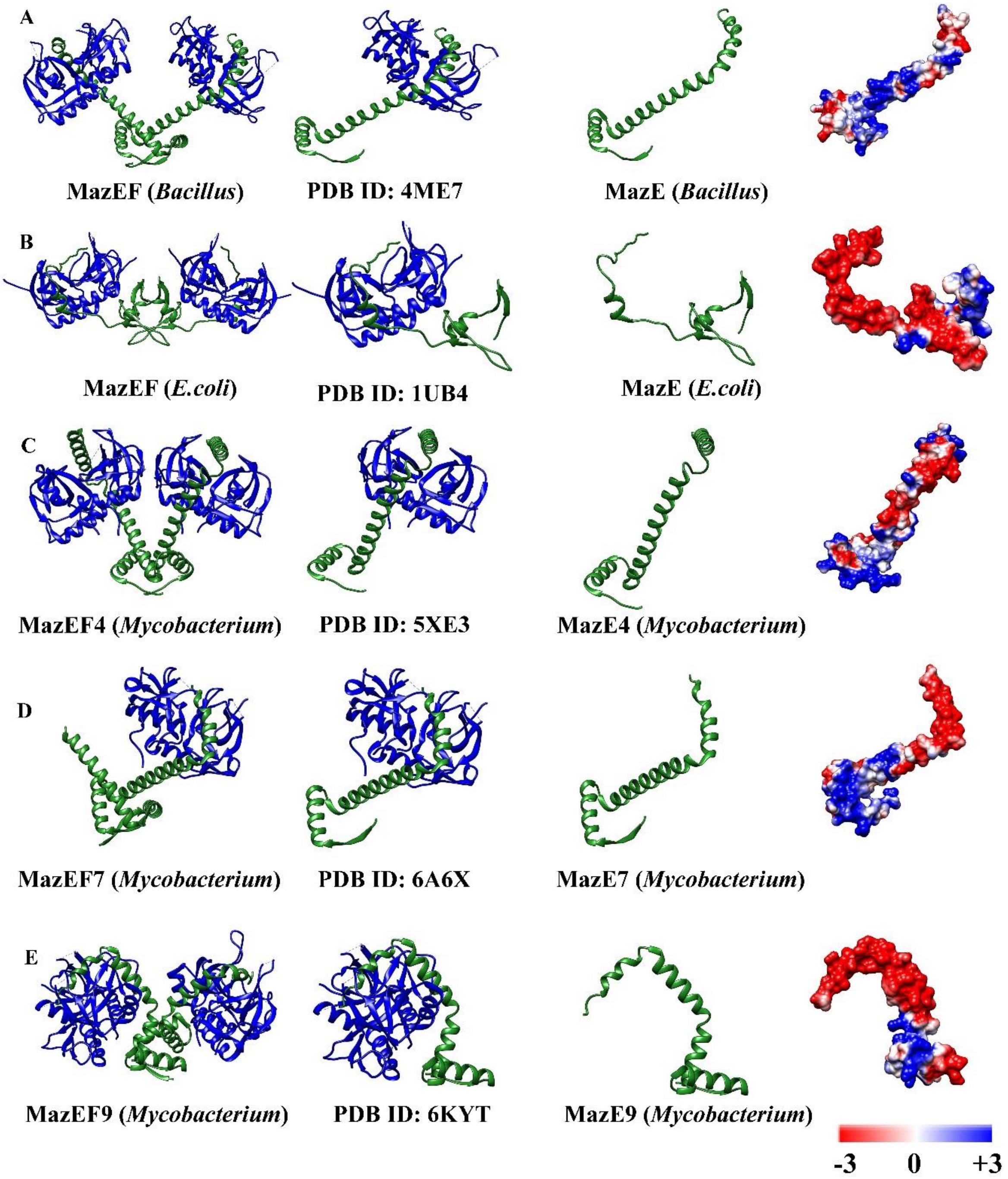

Figure S3. Relative antitoxin orientations in crystallographic structures of MazEF complexes. The first and second panel shows, the crystal structures and a single subunit of five MazEF TA complexes (A) MazEF (*Bacillus subtilis*), (B) MazEF (*Escherichia coli*), (C) MazEF4 (*Mycobacterium tuberculosis*), (D) MazEF7 (*Mycobacterium tuberculosis*), (E) MazEF9 (*Mycobacterium tuberculosis*), were used to understand the toxin interacting regions of the IDP antitoxins. In all cases, the toxins are shown in blue, and the antitoxins are shown in

green. The third and fourth panel shows the antitoxin alone (with the toxin chains removed) and the charge distribution of the antitoxin respectively. Except for *E.coli* MazE, other MazE antitoxins are largely helical upon toxin binding. The experimental data from our study, also indicate significant helicity for the MazE6 antitoxin. For regions involved in toxin binding, in the case of MazEF9 both the N-terminus and the C-terminus are involved in toxin binding. In the remaining cases the C-terminus is partially involved in interaction and the N-terminal is not involved at all. In contrast, our experimental data show that a significant part of the N-terminus and the initial part of the C-terminus is involved in toxin binding.

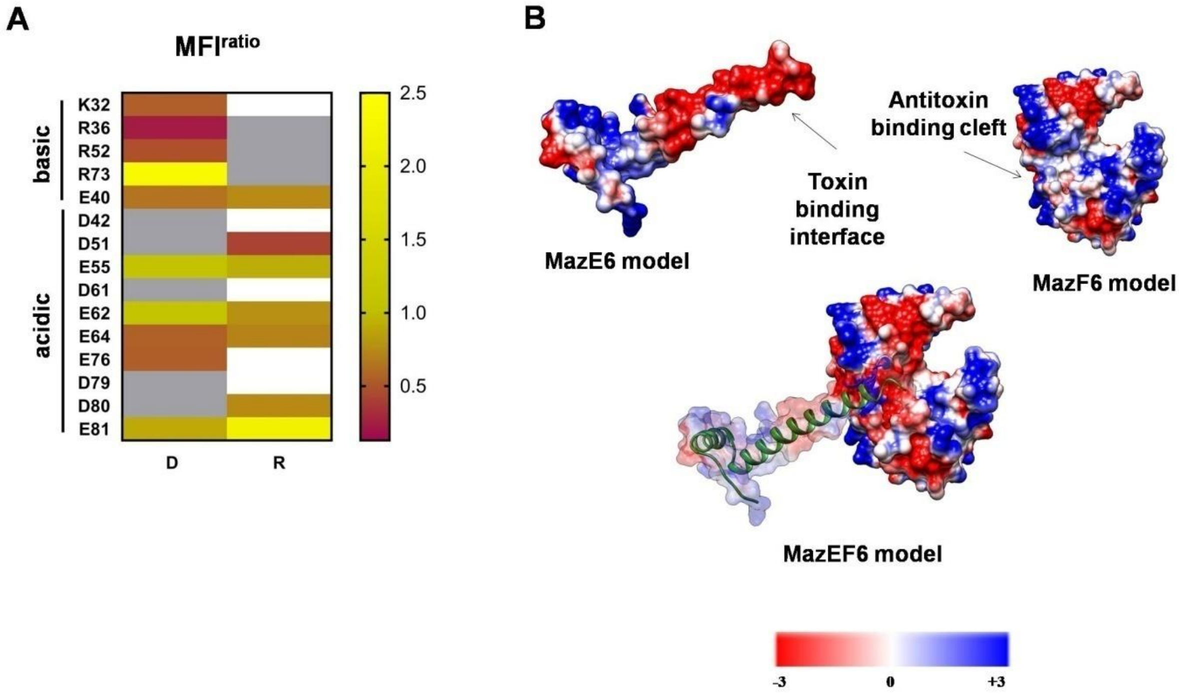

Figure S4. Charge distribution and charged mutational effects in MazE6. (A) Heatmap showing mutational effects at MazE6 positions bearing wildtype charged residues grouped based on the basic and acidic nature of their side chain groups. The MFI^{ratio} has been used to represent changes in toxin binding activity experimentally observed upon mutations to aspartate and arginine (B) Coulombic charge distribution of the MazE6 and MazF6 antitoxin structures modelled using SWISS-MODEL (Waterhouse et al., 2018) and visualised using Chimera software (Pettersen et al., 2004). SWISS-MODEL modelled only residues 58-78 of the antitoxin interacting with the cognate toxin.

```
001 Input_protein_seq              MKTAISLPDETFDRVSRRASELGMSRSEFFTKAAQRYLHELDAQL-LTG
002 UniRef90_A0A7I7MT07_1_82       MKTAISLPDETFDRVSRRASDLGMSRSEFFTRAAQRYLDELDAQA-LTG
003 UniRef90_X8AV83_1_82           MKTAISLPDETFDRVSRRASELGMSRSEFFTRAAQRYLDELDAQS-LTG
004 UniRef90_A0A1W9ZV28_1_82       MKTAISLPDETFDRVSQRARDLGMSRSEFFTRAVQRYLDELDAQS-LTG
005 UniRef90_A0A502EAL7_1_82       MKTAISLSDETFERVSRTASDLGMSRSEFFARAAQRYLDELDAQS-LTG
006 UniRef90_A0A1X0GDX4_1_82       MKTAISLPDETFDRASRRASDLGISRSEFFARAAQRYLDELDSES-LTG
007 UniRef90_UPI0008323BD8_1_82    MKTAISVSDETFDRVSRTASELGMSRSEFFARAAQRYLDELDAQS-LTG
008 UniRef90_A0A386UH06_1_86       MKTAISLPDETFDRASRRARDLGMSRSEFFTRAAQRYLDELDSQS-LTG
009 UniRef90_A0A2G5PDZ8_1_82       MKTAISVPDDTFARVSRTSNELGMSRSEFFSRAAQRYLDELDAQS-LTG
010 UniRef90_A0A3D0NH79_1_82       MKTAISVPDETFDRVCRRAVALGMSRSEFFARAAQRYLDELDAES-LTD
011 UniRef90_A0A5P8ZM98_1_82       MKTAISLPDETFERVTRRANDLGMSRSEFFTRAAQRYLDELDALS-LTE
012 UniRef90_A0A1E7KLL9_1_82       MKTAISLPDETYERASRRASDLGVSRSEFFARAAQRYLDELDAQS-LTG
013 UniRef90_UPI001621E97E_1_82    MKTAISVPDETFDRASRRAKELGLSRSQFFTRAAVNYLDELDAHS-VTQ
014 UniRef90_A0A2W6AMZ6_1_82       MKTAISVPDETFDRASRRASDLGMSRSEFFTQAAQRYLDQLDAHS-LSR
015 UniRef90_UPI0003B674CF_1_82    MKTAISLPDETFDRASRRARDLGMSRSEFFARAAQRYLEELDAES-VTQ
016 UniRef90_UPI0018CBBEE8_1_81    MKTAISLPDETFDRASRRAKELGMSRSEFFARAAVNYLDELDAKS-LTA
017 UniRef90_A0A4Y9P7B6_1_82       MKTAISLPDDTFERASRRALELGMSRSEFFARAAQRYLEELDAQS-LTH
018 UniRef90_A0A7W1ZG21_1_82       MKTAISVPDDTFDRASRRAVALGMSRSEFFARAAQRYLDELDAAS-VTH
019 UniRef90_A0A1S2WJL2_1_82       MKTAISLPDETFDRVSRRAADLGMSRSEFFARAAEHYLDELDARS-LSR
020 UniRef90_A0A1X0DS09_1_80       MKTAISVPDETFDRASRRAADLGMSRSEFFARAAEHYLDELDARS-LSR
021 UniRef90_A0A1Q7BZK4_1_81       MKTAISIPDETFERAEERAASLGMSRSEFFTRAAQRYLDQLDEES-LTG
022 UniRef90_UPI0018C9C051_1_81    MKTAISLPDETFDRASRRALELGMSRSEFFARAADRYLNELDAVS-VTK
023 UniRef90_A0A7Y0H587_1_82       MKTSISVPDETFEKATRRAHDLGMSRSEFFTRAAARYLEELDAES-TTR
024 UniRef90_UPI000D0E1F8C_1_80    MKTAISVPDETFNRASKRAAELGMSRSEFFTHAAQQYLEHLDASV-ITE
025 UniRef90_UPI001AAFE5BD_1_81    MKTAISIPDETFEKASRRAHDLGMSRSEFFTRAAAHYLDELDAES-VTR
026 UniRef90_A0A7V9GWH4_1_82       MKTAISVPDETFDRASRRAKELGLSRSEFFARAANSYLDELDAHS-VAR
027 UniRef90_A0A1Q3Q2K4_1_82       MKTAISLPDETFERASHRAGDLKMSRSEFFARAAERYLDELDAES-LTE
```

```
028 UniRef90_A0A354SBF1_1_82       MKTAISVPDETFEKATRRAHELGMSRSEFFSRAAVRYLDELDAES-VTR
029 UniRef90_A0A7Y0GVJ0_1_82       MKTAISVPDETFEKATRRAHDLGMSRSEFFSRAAARYLEDLDAES-VTR
030 UniRef90_A0A6V8KUB7_1_81       MKTAISIPDETFQRVERRATALGMSRSEFFVRAAQRYLDQLDAES-LTD
031 UniRef90_UPI0015F976F2_1_89    MKTAISLPDDTYDRATRRAGELGLSRSEFFARAALRYLDELDARS-LTA
032 UniRef90_A0A543P1M1_13_93      VKTAISIPDDTFAEVERRAKELGLNRSQFFTRAVQRYLADLESES-ITA
033 UniRef90_A0A1H2LAK4_1_81       MKTAISLPDATFERATRRAAELGMSRSEFFARAAEQYLRQLDDAS-LTA
034 UniRef90_A0A329QSX4_1_81       MKTAISVPDSTFERVSKCAARLGISRSEFFASAAERYLEQVEAAT-VTE
035 UniRef90_UPI00048133FC_1_82    MKTAISVPDEIYHQATRRARALGMSRSEFFARAADAYLHQLDARS-ITE
036 UniRef90_A0A0Q1HPB5_1_74       MKTAISIPDETFNEAERRAAALGVSRSEFFTTAARRYIEELDAQS-LTE
037 UniRef90_UPI00188AF3F7_1_84    MKAAISVPDETFDRVDRRARELGVNRSQFYTMAAERWLVELDSVE-ITA
038 UniRef90_UPI00146F4C5C_1_82    MKTAISLPDDTFARVEDAAGRLGVSRSEIFTRAAERYLDELDADS-ITQ
039 UniRef90_A0A7W9GU46_1_72       MKTAISLPDETFERATQRAAELGMSRSEFFARAAEQYLRHLDDAS-LTA
040 UniRef90_A0A4R4RMT7_1_73       MKTAISLPDETFERATQRAAELGMSRSEFFARAAEQYLRQLDDAS-LTA
041 UniRef90_A0A7Y3LFH0_1_82       MKTAISMPDETFQRAEREAKKHGMSRSEFFTKAAVRYLDELETES-LTH
042 UniRef90_A0A2G6JTD4_1_80       MKTAISVPDDTFAAASKRAQALGMSRSEFFTVAARRYLEELDRES-ITA
043 UniRef90_A0A542DGX5_1_81       MKTAISVPDETFAQVERGAKCLGVTRSEFYAKAAQFYLDHLERES-LTN
044 UniRef90_A0A1G9XC97_1_82       MKTAISIPDETFAEVERAAKCLGVTRSEFYARAARYYLEHLEQES-LTN
045 UniRef90_UPI001064113E_1_81    MKTAISVPDGTFAQVEQCAGELGVSRSEFYTRAAQYYLDHLRAES-LTS
046 UniRef90_A0A2T0LWM2_1_81       MKTAISIPDETFAQVERGAQGLGITRSEFYARAARFYLEHLERES-LTN
047 UniRef90_A0A6L5F2V1_1_79       MKTAISLPDDTFDEATKWARELGMSRSEFFALAARRYLDELDALS-VTE
048 UniRef90_A0A6N7Z5I7_1_81       MKTAISMPDETFEAATRQAASLGLSRSEFFTRAVRRYLDQLAAES-ATA
049 UniRef90_UPI0004173011_1_80    METAISVPDTTFEKVEQRATALGVSRSEFYTRAAQRYLADLETQD-LSD
050 UniRef90_UPI0007809952_1_78    MKTAISLPDAVFEAVTTRAAALGMSRSEFFSVAAQRYLRDLDEAA-LTG
051 UniRef90_A0A7V9ZI20_1_75       MKTAISLPDELFAEATRRAAELGISRSEFFSAAARRYLDDLSRES-LTG
052 UniRef90_A0A2W6G2J0_1_80       MKTAISVPDDTFEQASKQAAELGISRSEFFARAARRYLDELASRS-LTQ
053 UniRef90_F8B0B6_10_80          MKTAISIPDDTFDEVERHAVALGVSRSEFFTTAARHYLQKLDAQS-LTE
054 UniRef90_A0A543C8I6_1_82       MKTAISLPDEVFEGVERGAAELGISRSEFFARAAARYIAELESES-LTE
055 UniRef90_A0A5B2W4W7_1_82       MKTAISVPDETFAQVERSAKCMGVTRSEFYARAARFYLEHLEEES-LTS
056 UniRef90_A0A1W2FI02_1_79       MKTAISVPDETFVQVEQSAKSLGVTRSEFYTRAARFYLDHLEQQS-LTN
057 UniRef90_UPI0007802CDE_1_78    MKTAISLPDPVFEAVTTRAAALGMSRSEFLSVAAQRYLRELDEVA-LIG
058 UniRef90_UPI0007816E95_1_79    MKTAISVPDPIFDQVTRRAAELGVSRSEFFATAARRYLRELDDAG-VTA
```

```
059 UniRef90_UPI001563284D_1_81   MKTAISVPDETFAQVEQRAKRLGVTRSEFYARAARFYLEHLEQQS-LTN
060 UniRef90_A0A4P6Q602_1_81      MKTAISIPDEIFERVEQRAADLHVSRSEFFSRAAQSYLDELDSAE-LSR
061 UniRef90_A0A4D4J555_1_82      MKTAISVPDETFARVEQTAKCLGLTRSEFYARAARYYLDHLEEQS-LTE
062 UniRef90_A0A421B0D7_1_82      MKTAISVPDETFAQVDRGAKCLGVTRSEFYVRAAQYYLSHLEQES-LTN
063 UniRef90_A0A4T2C4P8_1_85      MKTAISVPDETFHRVDQRAAELHMNRSEFFSRAAERYLDALDALS-LTR
064 UniRef90_A0A7K0D2F8_1_81      MKTAISVPDDTYAKIERTVHSLGVTRSEFYVHAAQYYIAHLEETT-LTS
065 UniRef90_UPI000F78EFD4_1_85   MKTAISVPDATFKRIESRAKALGMSRSEFFARAAEHYINELDEHD-LTI
066 UniRef90_A0A7K0LN67_1_81      MKTAISVPDFIFSEVERRASELGVSRSEFYSTAARLYLNQLDSAS-LTQ
067 UniRef90_A0A6N7FTQ3_1_81      MKTAISVPDTTFERVEEYAAHSGMSRSEFYTKAAQRYLDELESEE-LSE
068 UniRef90_UPI000781CD88_1_76   MKTAISLPDPVFEAVTARARLLGVSRSEFLAVAARRYLRELDEQA-LTG
069 UniRef90_UPI001670FA12_1_79   MKTAISVPDETFAQVERSAKGLGLTRSEFYSRAARYYLEHLEQQS-LTN
070 UniRef90_UPI0007812280_1_77   MKTAISLPDPVFEAVTARAAALGVSRSEFLSIAAQRYLHDLDASS-VTA
071 UniRef90_A0A0N9IAA0_1_82      MKTAISVPDETFAQVDRSAKSLGVTRSEFYARAARFYLEHLEQES-LTN
072 UniRef90_UPI0019522ED5_1_80   MKTAISVPDVTYAKVERCVALLGISRSEFYTTAAQRYLEHLESRE-LTR
073 UniRef90_A0A4Y9NAF8_9_92      VKTAISIPDETFERVETSVAELGISRSEFFARAAKKYLDELESES-ITA
074 UniRef90_A0A653PKB9_25_109    VKTAISLPDELFTRVEARAAELGVNRSEFFAAAASRYLDETDAAG-LTD
075 UniRef90_UPI0016710A34_1_81   MKTAISVPDDTFAEVEDKVRNLGLTRSEFYVRAARHYLAHLEEHS-LTD
076 UniRef90_A0A1X3P978_1_80      MKTAISVPDGTFERAERVALRHGMNRSQFYAKAAERYADELDAHD-TTA
077 UniRef90_UPI0007806280_1_73   MKTAISLPDPVFEAVTARAKLLGVSRSEFLARAAQRYLRELDDHG-LTG
078 UniRef90_A0A511JC02_1_82      MKTAISVPDDTFSAVEARADELGMSRSEFFTRGAELLLERTAGDS-VRE
079 UniRef90_H6RM56_3_84          VKTAISVPDHILEQVNRKARDLGLNRSQFFSQAAERYLREID-DD-ITE
080 UniRef90_UPI001584AA74_1_59   MKTAISIPDSSFERVRYHAERLGMSRSEFFTKAAQRWADELDDQN-LTE
081 UniRef90_A0A7W7CLE4_7_87      -KTAISVPRETFLQVEVYADRLGVTRSEFFTRAARFYLEHIDEES-LTN
082 UniRef90_UPI00038171C2_1_69   MKTAISVPDETYSEVERHAQSLGVSRSEFYTRAARFYLDHLDRQS-LTE
083 UniRef90_UPI0012ED2160_1_73   MKTAISVPDATFHRVDKKAAELGLNRSEFYSLAAERYLRELEADD-LTT
084 UniRef90_A0A1S7BDR4_1_85      MKTAISLPDELFTRVEARAAELGVNRSEFFAAAASRYLDETGAAG-VTA
085 UniRef90_A0A521MTF4_1_68      MKTAISVPDATYSRVDKKAAELGMSRSQFYSVAAERYLRELESED-LTS
086 UniRef90_A0A7V8Y299_1_70      MKTAISVPDPVFERVKQHAERLGISRSEFFSTAAQRLADELEGDE-LTA
087 UniRef90_UPI0015DEDE4D_1_79   MKTAISIPDATFDRVEKRAAAMHLSRSEFYARAAQRYLEELESSE-LAQ
088 UniRef90_A0A7J9V7I4_1_81      MKTAISIPDATFEQVEEYAAASGMSRSEFYTKAAQHYLEDLETED-LSE
089 UniRef90_A0A4Q7KLD5_1_84      MKTAISVPDDTFSRAEERAAQLGISRSEFFARAAEDYLTRLDEHQ-LTE
```

```
090 UniRef90_A0A0Q5SIF2_1_85      MKTAISIPDATFERVERRVAELGINRSQFFARAAERYLDELEQRS-VTE
091 UniRef90_A0A2W6FAE8_1_79      MKTAISMPDVIYHQVTDRAATMGISRSEFFVHAARRYLDDLDAQS-DIE
092 UniRef90_A0A7V9LQV4_1_61      MKTAISLPNDTFEAATRRAAELGISRSQFFAVAARRYLEELDNTS-VIT
093 UniRef90_A0A7Y0M9N6_1_82      MKTAISMPDKTFERADALAKSLGLSRSEFISRAVDHYADELERSS-LTN
094 UniRef90_A0A2E8NZ72_1_66      MKTAISVPDPLFEKVERARPDLGMSRSQFYATAAQHYLDSVEREA-LTS
095 UniRef90_A0A7W1ASI9_1_71      MKTAISLPDDTFERVDRAARELGVSRSEFFARAAERWLAALEDGQ-TTA
096 UniRef90_A0A4R2JK17_1_76      ------MPDETFAQVERSAKCLGVTRSEFYARAARFYLEHLEQES-LTD
097 UniRef90_A0A7X5R350_1_66      MKTAISVPDNTFEAVEKQARLLNMNRSEFFSVAAQRYLDELNREQ-LTN
098 UniRef90_UPI00112C792A_1_81   MKTAISVPDEIFARVERVAGRHGMNRSQFYSAAAERYADELEDAD-LTA
099 UniRef90_A0A7V9J5T5_1_67      MKTAISVPDPVFARVERHAQRLGLSRSEFFAKAAARWADELEGGD-LTA
100 UniRef90_A0A3N2FTV2_1_81      MKTAISVPDSTYSAVEARAASLGISRSEFYTRGAQLLLERTDAAS-VTA
101 UniRef90_UPI00178ABCE6_1_81   MKTAISVPDEVFARVERVAALHGMNRSQFYATAASRYADELESSD-VTA
102 UniRef90_UPI0003B35733_1_72   MKTAISVPDATFARVDAAADRLGMSRSEFFSRAAERWLAALDDDG-TTA
103 UniRef90_UPI00058CFADA_1_81   MKTAISVPDDTFRRAEAAAGKHGMNRSQFYAAAAERYARELEGSD-LTA
104 UniRef90_A0A6N7EEV9_1_80      MKTAISLPDETFERAERAAKRLGITRSELYARAVDAFVESLDRTS-VTS
105 UniRef90_A0A138AXK4_1_82      MKTAISLPDDAAERFDRVADKHGMSRSEFYRRAAERYADELADAR-LTE
106 UniRef90_UPI00195976AE_1_81   MKTAISVPDDVFARVERVAARHGMNRSQFYATAASRYADELEAGD-LTA
107 UniRef90_A0A1A3P0A6_9_58      -------------------------------RGQRYLDELDAQS-LTG
108 UniRef90_A0A3N4ZMD4_1_82      MKTAISLPDHAAERFDHIAQKHGMTRSEFYRRAAEHYADELAGAD-LTA
109 UniRef90_UPI00047AB853_1_82   MKTAISLPDHAAMRFDRIAQKHGMTRSEFYRRAAEHYADELDGAE-LTA
110 UniRef90_A0A7L4YQG6_1_74      MKTAISVPDETFERIERVAKKHGINRSQFYATAAAKYADELESNE-LTA
111 UniRef90_N1V7L8_1_80          MNTTVSMPDETLRRADRAAQTLGVSRSEFVARAIERYADELERNS-LTN
112 UniRef90_UPI001782D03F_1_75   MKTAISVPEPTYSAVEARVEELGMSRSEFYTRGAELLLARTDAAT-VTA
113 UniRef90_F6FX19_1_77          MKTAISLPDSAAEHFDRVAKKHGMTRSEFYRRAAEHYVEELDGAD-LTA
114 UniRef90_A0A3G9J3E9_1_80      MKTAISVPDEIFERVERMAKRHGINRSQFYSQAAERYASELEAND-LKD
115 UniRef90_A0A0B0DCZ2_1_82      MKTAISLPDQAALRFDRIAQQYGMSRSEFYRRAAEHYADTLADAD-LTA
116 UniRef90_A0A7G5LYH4_1_69      MKTAISIPDPLFARIDAKAAEQGLSRSAFFATAAERYLDELSHDD-LTR
117 UniRef90_A0A7K0PL79_1_73      MKTAISVPGPTFARVDAAAARLGVSRSEFYARAAERWLLELERES-VTA
118 UniRef90_T0ZH80_1_60          -------------RVNRRASEMGMSRSEFFARAAERYLEQLDSAS-ITA
119 UniRef90_A0A1R1LB14_1_82      MKTAISLPDHAAERFDRVAHKHGMTRSEFYRRAAEQYADELADVD-LTA
120 UniRef90_A0A3E0VWF3_1_85      MKTAISVPDPLFRQVEARTAQLKISRSEFYSTAATHYLRELERTS-ITE
```

```
121 UniRef90_A0A2W6CSY0_1_71        MKTAISLPDRTFHRVNDAAARLGVSRSEFFARAAERWLAVLEDDG-TTE
122 UniRef90_A0A7V9QTF7_1_63        MKTAISMPDHTYEAVTRRAAELGISRSRFLVLAAEQKLAEEAADD-LTA
123 UniRef90_UPI00140D7277_1_82     MKTAISLPDDAAERFDDVARRHNMTRSEFYRQAAERYADTLSGGD-LTA
124 UniRef90_UPI001941C382_1_58     MKTAISIPDHEAERIDASARRHGMTRSEFYRRAAGRYADELDAQD-VTA
125 UniRef90_A0A562UY95_1_81        MKTAISMPDEVFRKVQECADELGVSRSEFITSAAERYVAYVRNHT-LTH
126 UniRef90_A0A0Q4UTL1_1_62        MKTAISVRDELFERVERAIAEHGMNRSQFYAAAAERYLSELADDS-ITE
127 UniRef90_A0A1X1RGN2_1_82        MKTAISLPDDAAERFDRIAQRHGMTRSEFYRRAAEQYADELADAD-LTA
128 UniRef90_A0A7C5J0X7_1_78        MKTAVSLPDEIFQRASKEAKRLGISRSELVARALREYLRDRSSRE-VRA
129 UniRef90_A0A0B1ZWM2_1_77        MKTAISVPDADFERFERVASRNGMSRSEFYRRAGARYADELEGTSALTA
130 UniRef90_UPI000D06493E_1_81     MKTAISLPDHAAGRFDDVARRHGMTRSEFYRRAAERYADELSEAD-LTR
131 UniRef90_A0A7Y9ICF6_1_81        MKTAISLPDQAAARFDRIARRHGMTRSEFYRRAAEQYAEDLADAE-LTA
132 UniRef90_A0A5Q6RY07_8_86        VKTAISIPDRLFERVDAEAAERGMSRSAFFAAAAEHYLAEIEHDD-VTR
133 UniRef90_A0A1S8CAB9_3_66        -----------------TAGRSRYSRSEFFATAARRYLDELDAAS-LTS
134 UniRef90_UPI00068C398D_1_74     MKTAISVPEETFALVESRAAELGISRSEFYTRGARLLLERTAADS-VQE
135 UniRef90_A0A7Z7NB59_5_60        ------------------------------RRAAQRYLDELDSQS-LTR
136 UniRef90_A0A6M5J1T1_1_73        MKTAISLPDDDFERFDRVASRHGMNRSEFFRAAASRYADELDGDKELTR
137 UniRef90_UPI00037D62DF_1_82     MKTAISLPDSAAQRFDRIAQRHGMTRSEFYRRAAEQYADNLADAD-LTA
138 UniRef90_A0A0U4NWH5_1_60        MKTAISLPDELFQRVEAARAEDGTGRSEFFAEAARAYLQQRESER-LRE
139 UniRef90_A0A3M9MCJ5_1_82        MKTAISLPDSEAERFDRVAQRHGMTRSEFYRRAAEQYADALADAD-LTE
140 UniRef90_A0A420X6M3_8_85        ---TIYLNDATAAHVDDAVQRFGLHTSEFFSKAAESYIDKLESQS-VTA
141 UniRef90_A0A345IFH3_1_65        MKTAISLTDELFEAVETNAKKLGVSRSQFFAMGAEKLIRQLEAEE-ITA
142 UniRef90_UPI00047DD988_1_75     MKTAISIPDGDFEHFERVAARHGMNRSEFFRRAGLRFVEELEGKAELTA
143 UniRef90_UPI0018CAEB06_1_70     MKTAISIPDRDFERFERVAGRLGMNRSEFFRLAASRLVEELEGEAELTA
144 UniRef90_A0A6I3MBG9_1_77        MKTAISIPDGDFERFERVAARHGMNRSEFFRRAGRRFVEELEGEAELTA
145 UniRef90_A0A2A9D1X6_8_86        MKVAISLPDEALRRIDDAAAALGQNRSEFFRTAGLKYAVEVEAGA-LTH
146 UniRef90_A0A7W4VTP9_1_81        MKTAISVPEEIYARAERAARKLGLNRSQFYSAAAERLAAEVESAD-VTA
147 UniRef90_A0A387BRJ4_1_80        MKTAISVPDDDFQRFERIAARNGMSRSEFYRRAGAKLADELEGASRLTA
148 UniRef90_UPI0009FEB7FE_1_75     MKTAISLPDADLERLDRVAARHGMNRSEFFREAGRRYADELEGASDLTA
149 UniRef90_UPI000932B65A_1_61     MKTAISLPDSVFEQAESLAQQLGMSRSELYTKALQAYLKKHDRAQ-ILI
150 UniRef90_UPI001359AD9E_1_81     MKTAISLPDEEFERFERVAKRHAMNRSEFFRRAGERLADELEGTSELTR
```

```
001 Input_protein_seq               IDRALES----IHGT-------DEAEA-LAVANAYRVLET--MDDEW
002 UniRef90_A0A7I7MT07_1_82        IDSALES----LRGT-------DESES-AAVAAGYRVLAA--VDDEW
003 UniRef90_X8AV83_1_82            IDMALEN----LHDT-------DEAEA-AAVTVGHRVLDA--VDDEW
004 UniRef90_A0A1W9ZV28_1_82        IDSALGR----LHGT-------DEAET-AAVAIGRRVLDA--VDDEW
005 UniRef90_A0A502EAL7_1_82        IDSALER----LDGT-------DEAEA-AAVAVGYRVLGA--VDDEW
006 UniRef90_A0A1X0GDX4_1_82        IDSALAC----LGGT-------DEAEA-AAVAGGHRVLDA--VDDEW
007 UniRef90_UPI0008323BD8_1_82     INSAMEH----LDRT-------DQAEG-AAVAAGRRVLDA--VDDEW
008 UniRef90_A0A386UH06_1_86        IDDALEHLGERLGDS-------DGGQA-AAVAVGHRVLDA--VDDEW
009 UniRef90_A0A2G5PDZ8_1_82        IDDAVAR----LSSA-------EDSAA-SAVAAGHSLLDD--MDDEW
010 UniRef90_A0A3D0NH79_1_82        INTALDA----VDSL-------DESQQ-AAVAVGRRVAGT--IDDDW
011 UniRef90_A0A5P8ZM98_1_82        INSALER----LVGP-------DDETA-DAVAAGHRVLHD--TADEW
012 UniRef90_A0A1E7KLL9_1_82        IDDALEY----VGTL-------DEATS-DAIEAGRRLLAD--TGDEW
013 UniRef90_UPI001621E97E_1_82     IDAALAH----CSGS-------DESNA-AAVEAAHGVLRN--SDDEW
014 UniRef90_A0A2W6AMZ6_1_82        IDSALER----LDGP-------DDSAT-LAVTSAHHRMTD--SNDDW
015 UniRef90_UPI0003B674CF_1_82     IDAALDR----IGAV-------DVASL-AAVEAGHRLLLD--APDEW
016 UniRef90_UPI0018CBBEE8_1_81     MDVALDL----IGKT-------DESTE-LAVSAGRRVLGA---TDEW
017 UniRef90_A0A4Y9P7B6_1_82        IDSALDS----LREG-------DDSGA-AAIGVGRRLMAG--TADEW
018 UniRef90_A0A7W1ZG21_1_82        IDAALKG----LAGP-------DGSTT-EAVASGRRLLAA--SDDQW
019 UniRef90_A0A1S2WJL2_1_82        IDSALEH----LGTP-------ADTSA-DAVAVGHRVLDA--AGDAW
020 UniRef90_A0A1X0DS09_1_80        IDSALEY----LGAP-------DETNA-DAVAVGHRVLDA--AGD--
021 UniRef90_A0A1Q7BZK4_1_81        IDAAIAL----VGD--------DDSAE-VAVAAGRRYLRA--AEDDW
022 UniRef90_UPI0018C9C051_1_81     IDEALAG----IDN--------DESSV-AAIEAGRRVLAG--GTEEW
023 UniRef90_A0A7Y0H587_1_82        IDLAVAA----LAGR-------DDSAA-DAVAVGQRVLDD--VSGDW
024 UniRef90_UPI000D0E1F8C_1_80     IDRTLGL----VDD--------DDSSA-VAAQAGRRFLS---RDDEW
025 UniRef90_UPI001AAFE5BD_1_81     IDAAVDA----LGV--------DDSAA-DAVAAGRRLLDA--EADDW
026 UniRef90_A0A7V9GWH4_1_82        IDAVLEQ----CGAL-------DGSNA-AAVEAAHGFVHA--SDDEW
027 UniRef90_A0A1Q3Q2K4_1_82        IDASLDV----VDDI-------GHDAE-VAVEAGRRTLAS--LDDEW
028 UniRef90_A0A354SBF1_1_82        IDQALGA----QAEQ-------DDSAA-DAVAVGHRLVAD--ASGDW
029 UniRef90_A0A7Y0GVJ0_1_82        IDLAVAA----LAER-------DDSAA-DAVAVGRRVLAD--ASGEW
```

```
030 UniRef90_A0A6V8KUB7_1_81      INAALDL----AGD--------DDTSR-AAVEAGWHRLAA--DQDEW
031 UniRef90_UPI0015F976F2_1_89   IDEALDT----VAAAGAGDAEGDSGSG-DAIAAGRAVLGA--LDDEW
032 UniRef90_A0A543P1M1_13_93     IDAALEA----AGE--------DDSNA-WAAAAGRRMLAR--TDDDW
033 UniRef90_A0A1H2LAK4_1_81      IDAALDR----GGD--------DDSAG-AAVAAGRRTLTA--AESDW
034 UniRef90_A0A329QSX4_1_81      INRALAA----TGT--------DDSNE-LAALAGTSRLEA--SDDEW
035 UniRef90_UPI00048133FC_1_82   INEAIGQ----QTGI-------DDSLA-AAVAGGRATLER--NTDEW
036 UniRef90_A0A0Q1HPB5_1_74      IDAALEL----AGP--------DDSFE-AAARAGRRAL---------
037 UniRef90_UPI00188AF3F7_1_84   LDNAVEA----AGPSGR-----AEAAQ-FAAGAARSLATL--SDDEW
038 UniRef90_UPI00146F4C5C_1_82   LDAALEA----AGGP-------DASNR-EAAEHGRSVLAA--TDEEW
039 UniRef90_A0A7W9GU46_1_72      IDAILDR----TGD--------DDSAS-AAVAAGRG-----------
040 UniRef90_A0A4R4RMT7_1_73      IDEILDR----TGD--------DDSAA-AAATAGRRA----------
041 UniRef90_A0A7Y3LFH0_1_82      IDQVIDT----QVQS-------DDSTL-DAVNVGHRLLAG--MDEDW
042 UniRef90_A0A2G6JTD4_1_80      INDALRT----AGA--------DDSAA-AAAAAGRRYLR---FQDEW
043 UniRef90_A0A542DGX5_1_81      IDSALEL----IDD--------DDSSA-VAVTAGRRRLEL--ADDEW
044 UniRef90_A0A1G9XC97_1_82      IDAALEV----IGDE-------DDSAA-AAAGAGRRVLAQ--VDDEW
045 UniRef90_UPI001064113E_1_81   IDAALAM----IGE--------DDSAE-AAVAAGRHRLAQ--TDDEW
046 UniRef90_A0A2T0LWM2_1_81      INSALEL----IDG--------DDSSS-AAVIAGRRRLEL--ADDEW
047 UniRef90_A0A6L5F2V1_1_79      VNEALAA----AGD--------DDSAA-AAVAVGRRRLLD--EDD--
048 UniRef90_A0A6N7Z5I7_1_81      IDEALAL----AGS--------DDSNA-AAASAGHVRLAA--GDDDW
049 UniRef90_UPI0004173011_1_80   IDDALAR----IQG--------EDSST-LAVRAGHRRIAE--LSGA-
050 UniRef90_UPI0007809952_1_78   IDSVLER----IAIAE------DESTA-FAAQVGRGLLES-------
051 UniRef90_A0A7V9ZI20_1_75      INQVLDL----LDYD-------EEASV-VAVEAGRRRL---------
052 UniRef90_A0A2W6G2J0_1_80      VNEALRV----AGS--------DDSAA-AAAQSGRQRLAA---GDEW
053 UniRef90_F8B0B6_10_80         IDAALAL----AGP--------DDSSE-AAVRAGR------------
054 UniRef90_A0A543C8I6_1_82      INAALDA----VGED-------KDLIR-DVTAQARRTFLA--MDDDW
055 UniRef90_A0A5B2W4W7_1_82      INAALEL----IGDD-------DDSAA-TAVDAGRRVLAL--ADDEW
056 UniRef90_A0A1W2FI02_1_79      INAALDG----V----------DESSE-VAVAAGRRHLAS--ADDEW
057 UniRef90_UPI0007802CDE_1_78   IDSVLER----IASAE------DESTA-LALDAGRTLLER-------
058 UniRef90_UPI0007816E95_1_79   IDSVLER----VAIAQ------DESTR-FVADAGRALFDRE------
059 UniRef90_UPI001563284D_1_81   INEALDL----IGD--------DDSGA-AAAGAGRRHLAG--ADDEW
060 UniRef90_A0A4P6Q602_1_81      IDEAIAL----SGE--------DDSNA-AAAAAGRRSILD--ASGEW
```

```
061 UniRef90_A0A4D4J555_1_82      INAALER----VGDD-------EDSAA-AAAAAGRRRLAR--TDDEW
062 UniRef90_A0A421B0D7_1_82      INAALDV----IGAD-------DDSGA-AAVTAGRGVLAQ--ADDEW
063 UniRef90_A0A4T2C4P8_1_85      IDAAVLR----SGTLL------QAETA-TVTRVGRERLAALTADDEW
064 UniRef90_A0A7K0D2F8_1_81      IDAALDL----ISGT-------DDSNA-AAASAGRRLLAE---SDEW
065 UniRef90_UPI000F78EFD4_1_85   INEALER----GGDTV------EAESR-EFAELGRAHLAGLTADDEW
066 UniRef90_A0A7K0LN67_1_81      IDDLLER----IEP--------DDSSE-VAVVHGRATLGA--GDDGW
067 UniRef90_A0A6N7FTQ3_1_81      INEAIAL----VGE--------DDSND-AAAAAGRRSIAA--LSGDW
068 UniRef90_UPI000781CD88_1_76   IDSVLER----IAVAE------DESTR-FVAEAGRGLL---------
069 UniRef90_UPI001670FA12_1_79   INAALEL----VGD--------DDSAS-SAVGAGRQVLA----DDEW
070 UniRef90_UPI0007812280_1_77   IDSVLER----VALAE------DESSR-FARDAGRRLLE--------
071 UniRef90_A0A0N9IAA0_1_82      INDALDV----IGSA-------DDSGT-AAAAAGRHRVAG--SDDEW
072 UniRef90_UPI0019522ED5_1_80   IDEALDL----AND--------DDSAA-AAVAAGRRRLAL---DEED
073 UniRef90_A0A4Y9NAF8_9_92      INAAIDA----GAVD-------EDLDR-DVTEHGRRMLLELTADDEW
074 UniRef90_A0A653PKB9_25_109    VDTALRR----AGVAS------AEEAR-GVAAAGRDRLAELTAGDDW
075 UniRef90_UPI0016710A34_1_81   IDAALDL----AGD--------DDSGA-VAAAAGRRALAM--SDDEW
076 UniRef90_A0A1X3P978_1_80      IDSVVDV----VNA--------DESAR-FAVASGHRMADG---DDEW
077 UniRef90_UPI0007806280_1_73   IDSVLER----VAVAE------DESAR-FVLETGR------------
078 UniRef90_A0A511JC02_1_82      IDLVVEY----VGAA-------DASQS-AAVAAGRRVLEA--DGDDW
079 UniRef90_H6RM56_3_84          INAALDA----AGD--------DDSNA-VAAAHGRRMLLELTADDEW
080 UniRef90_UPI001584AA74_1_59   IDEALDR----LDP---------------------------------
081 UniRef90_A0A7W7CLE4_7_87      INAALAY----VEGP-------DTTTT-AAVAAGRRHLAG--MADEW
082 UniRef90_UPI00038171C2_1_69   INAALAV----AGA--------DDSAG-VAVRA--------------
083 UniRef90_UPI0012ED2160_1_73   LDDALAR----SGHAA------ESEAK-DFAAHAR------------
084 UniRef90_A0A1S7BDR4_1_85      VDTALRH----AGVAS------AEEAR-VVASAGRDRLAAVTVGDDW
085 UniRef90_A0A521MTF4_1_68      INEALAR----SGDAA------RSEAL------------------EW
086 UniRef90_A0A7V8Y299_1_70      IDRALEH----VGP--------EDASE-FVTAAA-------------
087 UniRef90_UPI0015DEDE4D_1_79   IDEAVSL----AEE--------DDSNT-NAATAGRTRLTE--ASG--
088 UniRef90_A0A7J9V7I4_1_81      INQAIAL----AGE--------DDSTV-SAVAAGRRSLAD--LSGDW
089 UniRef90_A0A4Q7KLD5_1_84      IDEAVAL----IADHPE----GADSNE-DAVRAGHAVLAA---NDEW
090 UniRef90_A0A0Q5SIF2_1_85      LNAAIAL----DAGRS------DSETA-DFVRFSRRRLESATADDAW
091 UniRef90_A0A2W6FAE8_1_79      INAAVDP----A----------EDSNA-VAAAAGRRRLER--VEDEW
```

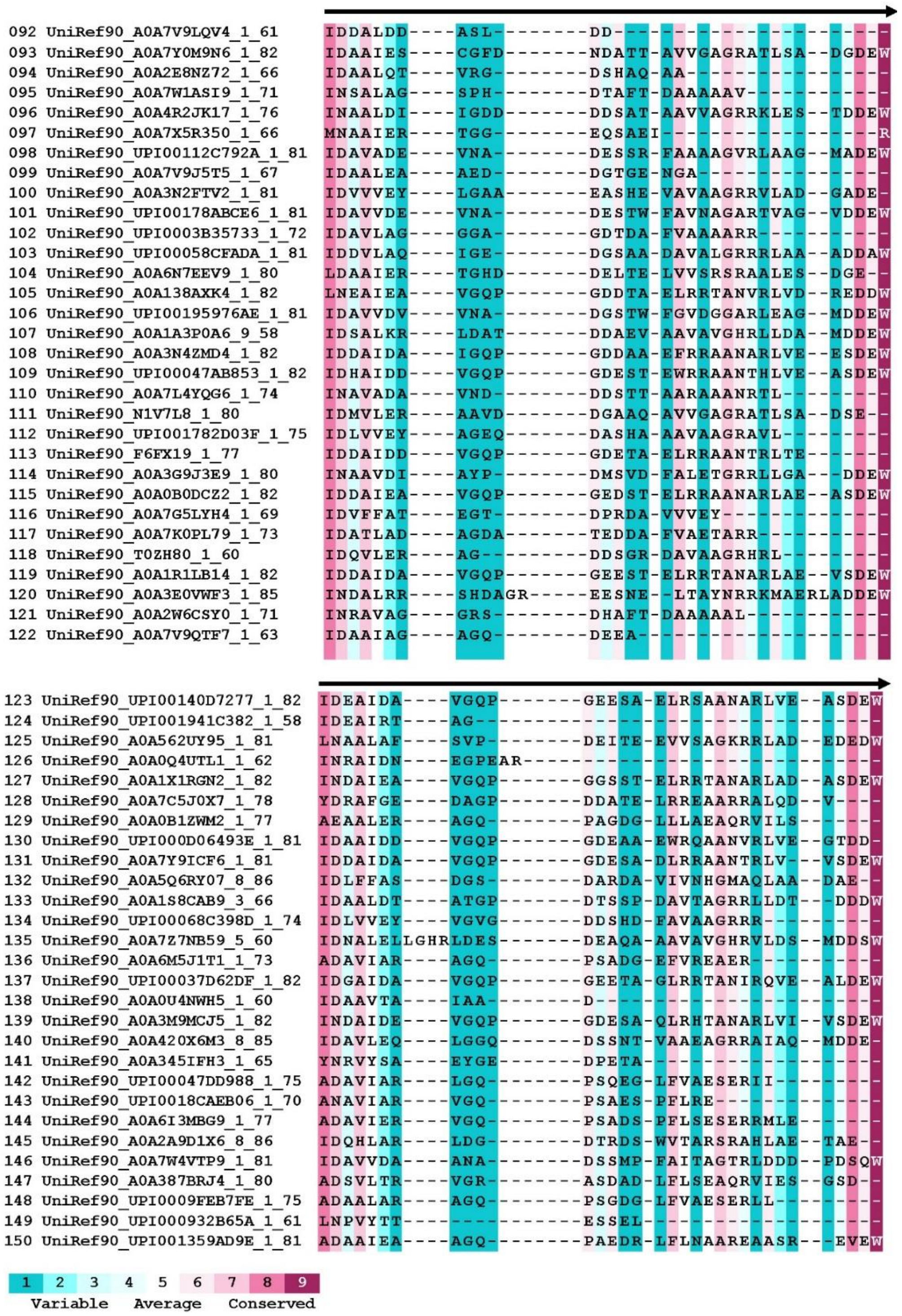

Figure S5. The MSA of 150 homologous sequences with the MazE6 antitoxin. The alignment of the query MazE6 input sequence (001) with 150 other homologous sequences generated using ConSurf server (Ashkenazy et al., 2016) is shown. A minimal sequence identity of 20% was used to filter out the homologous sequences. The black arrow designates the residues of the input sequence which has been used for the experimental study.

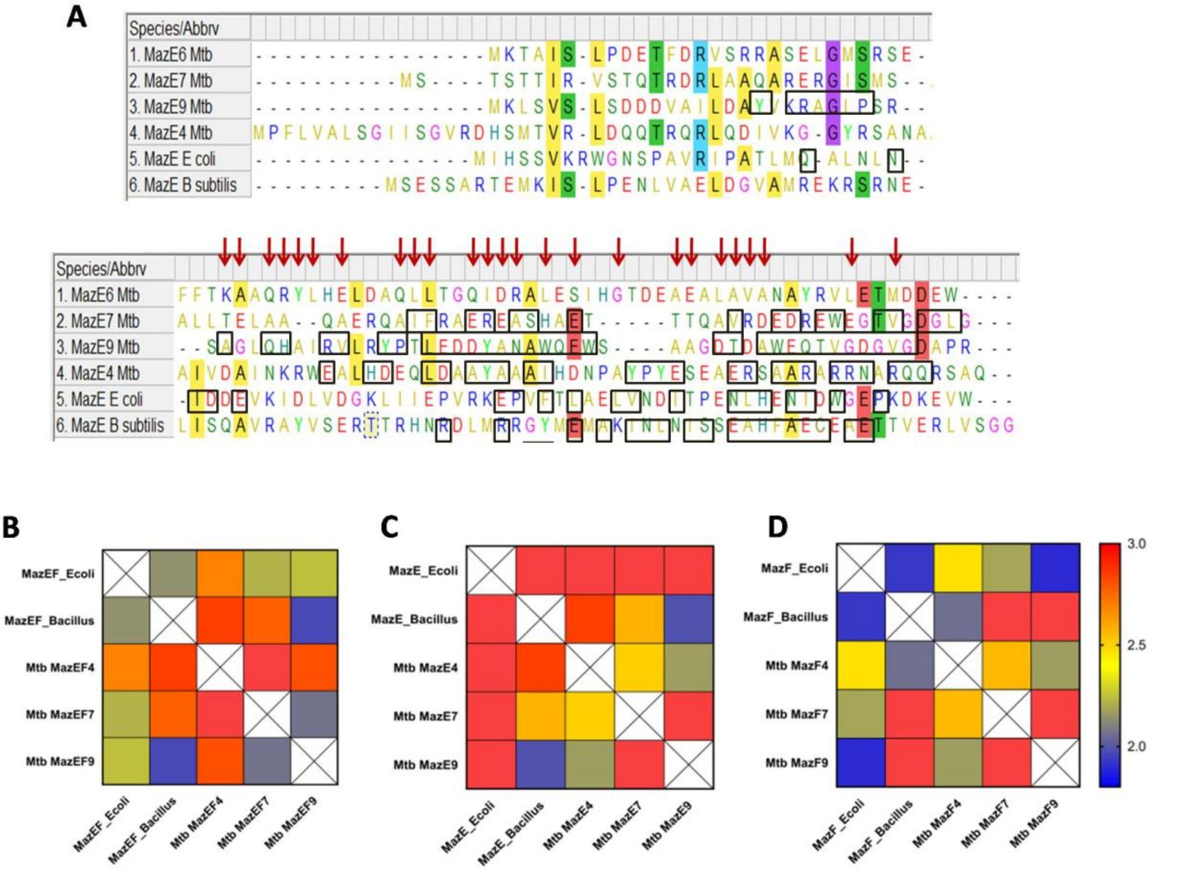

Figure S6. Multiple sequence alignment and structural comparison of MazE6 and its homologs for which the complex structures are known. (A) MSA for MazE6, MazE7, MazE9 and MazE4 homologs in *M. tuberculosis* and MazE homologs in *E. coli* and Bacillus subtilis. The MazE6 positions inferred to be functionally important for toxin binding from the present study are indicated by red arrows. In case of all other homologous sequences, the residues in contact with cognate toxin in the available complex structures are highlighted with black boxes. The positions with >50% sequence identity in the MSA have their background highlighted in red color. The inferred binding residues in MazE6 do not significantly overlap with those identified by crystallography in any of the other Maz homologs. (B-D) Heatmaps showing the root mean square deviations (in Å) for pairwise comparisons of the structures of the homologous MazEF complexes, MazE antitoxins and MazF toxins respectively obtained using MM-align server (Mukherjee and Zhang 2009).

Supplementary Tables

Table S1. List of MFI^{ratio} values of Asp and Arg mutants for the two biological replicates along with their Mean and Standard deviation. MFI^{ratio} values of each mutant are normalised to the WT values for each replicate.
